# UK Kidney Association Clinical Practice Guideline on vascular access for haemodialysis

**DOI:** 10.1186/s12882-025-04374-y

**Published:** 2025-08-14

**Authors:** Emma Aitken, Hameed Anijeet, Damien Ashby, Wayne Barrow, Francis Calder, Brett Dowds, Catherine Fielding, James Gilbert, Rob Jones, Narayan Karunanithy, Zaib Khawaja, Emma Roberts, Mike Robson, Rukshana Shroff, Hannah Stacey, Peter Thomson, Dan Waters

**Affiliations:** 1 Queen Elizabeth University Hospital, Glasgow, UK; 2 Royal Liverpool University Hospital, Liverpool, UK; 3 Hammersmith Hospital, London, UK; 4 Patient representative, Bangor, UK; 5 Guy’s & St Thomas’ NHS Foundation Trust, London, UK; 6 University Hospitals of Derby and Burton, Derby, UK; 7 New Foscote Hospital, Banbury, UK; 8 Queen Elizabeth Hospital, Birmingham, UK; 9 Betsi Cadwaladr University Health Board, Bangor, UK; 10 UCL Great Ormond Street Hospital Institute of Child Health, London, UK; 11 Addenbrooke’s Hospital, Cambridge, UK; 12 King’s College, London, UK

**Keywords:** Haemodialysis, Vascular access, Fistula, Graft, Vascular catheter

## Abstract

**Abstract:**

This guideline is written primarily for doctors and nurses working in dialysis centres and related areas of medicine in the UK, and is an update of a previous version written in 2015. It aims to provide guidance on how to provide vascular access care for patients approaching and undergoing haemodialysis, and provides a standard of care which centres should in general aim to achieve. We would not advise patients to interpret the guideline as a rulebook, but perhaps to answer the question: “What does good quality vascular access care look like?”. The guideline is split into sections: each begins with a few statements which are graded by strength (1 is a firm recommendation, 2 is more like a sensible suggestion), and the type of research available to back up the statement, ranging from A (good quality trials so we are pretty sure this is right) to D (more like the opinion of experts than known for sure). After the statements there is a short summary explaining why we think this, often including a discussion of some of the most helpful research. There is then a list of the most important medical articles so that you can read further if you want to – most of this is freely available online, at least in summary form.

A few notes on the individual sections:

1.  This section covers key concepts relevant to vascular access and focusses on access type selection, including a historical introduction and review of the key literature informing our understanding. This explains why we are moving away from the outdated advice in previous guidelines (e.g. that ‘all patients should dialyse with a fistula as first choice’) towards a process which treats dialysis access selection as a choice, respecting patient individuality, aiming to provide high quality assessment and advice, so that patients are supported in making informed decisions. The basic concept of the fistula as optimal access is highlighted and remains valid, but it is placed within a more modern concept of care, in which the patient is at the centre of the decision process.
2.  This section addresses the initial planning of access, from education and vein preservation, through to the timing of assessment and access formation, emphasising in particular the need to plan ahead.
3.  This section deals with the formation and routine care of AV access (fistulas and grafts), covering access type and configuration, surgical and anaesthetic technique, the maturation period (before a fistula is ready to be used), and initiation and maintenance of optimal cannulation (needling).
4.  This section deals with some of the complications of AV access. Research in this area is ongoing and not yet sufficient to give clear guidance, so we emphasise again the importance of involving patients in treatment decisions.
5.  This section deals with the placement and routine care of catheter access (lines), covering location, technique, anticoagulant locks, and regular exit site disinfection and dressings.
6.  This section deals with catheter complications, like infection and poor flow, which are sometimes life-threatening, and for which the catheter sometimes needs to be changed.
7.  This section deals with central venous stenosis (narrowing of veins deep in the chest) which is mostly a long term complication of catheters, but which is relevant to the planning of all types of access. We thought this important condition deserved its own section.

Most of the concepts relevant to adult patients apply equally to children and adolescents, so there is no separate Paediatric section, and unless stated, guidance applies to children as well as adults. Where they do exist, differences are highlighted within the statements and rationale, sometimes with separate paragraphs or subheadings. Access for peritoneal dialysis is not included in this guideline since it is covered elsewhere, and the guideline is not exhaustive, with several aspects not covered, though they may be addressed in future versions. The guideline’s principle focus is areas of mainstream practice for which there is variation across different UK centres, in general not covering newly developed or rarely practiced techniques, and it is not intended to replace handbooks and review articles. The guideline’s main anticipated audience is NHS professionals caring for patients who are receiving or planning haemodialysis, but it is written to be as accessible as possible to patients and carers also. There are appendices at the end which explain the meaning of words and concepts which are used throughout the guideline, especially the medical and statistical terminology.

**Clinical trial number:**

Not applicable.

## Background

Haemodialysis continues to expand in the UK with over 25,500 patients currently being treated, representing a 6% increase since publication of the previous Renal Association guideline for haemodialysis vascular access, and the patient group continues to develop: the typical patient is now 67 years old with a median history of 3.2 years on renal replacement therapy. The authors of this guideline aimed principally to update the previous guideline according to the latest research and experience, but also to expand the scope into areas not previously covered, but relevant to haemodialysis vascular access practice.

The guideline was written collaboratively: lead and co-authors for each section conducted literature reviews and wrote first drafts of the statements and rationale. Feedback and discussion were provided by all authors via email exchanges and meetings, revised versions were produced with editorial input from the chairs, and these were subsequently agreed by all authors. The author group was broad in professional representation, including experienced nurses, nephrologists, surgeons, and radiologists, and incorporated also one specialist registrar (pre-consultant doctor). Three current haemodialysis patients also co-authored the guideline, commenting on a number of aspects, and in particular giving advice on tone and readability.

Systematic literature searches were undertaken by lead authors to identify all relevant evidence published up until the end of December 2020. Compound search terms were used which included haemodialysis and vascular access identifiers, e.g. (hemodialysis(tiab) OR haemodialysis(tiab) OR dialysis(tiab)) AND (“vascular access”(tiab) OR fistula(tiab) OR CVC(tiab) OR “venous catheter”(tiab) OR AVF(tiab) or AVG(tiab)), followed by title/abstract-filtered topic terms, e.g. (decision(tiab) OR selection(tiab) OR choice(tiab)), followed by negative terms (e.g. to exclude animal studies), finally with date and language restrictions, e.g. (“last 10 years”(dp) AND english(lang)). Searches were conducted in MEDLINE, PUBMED, Embase, CINAHL, and The Cochrane Library, and supplemented with papers handpicked from the reference lists of review papers.

The strengths of the recommendations and the level of supporting evidence are coded as previously using the Modified GRADE system.

There are some limitations in scope, for example we have not covered infrastructure or workforce since these will be addressed separately by the UK Kidney Association in a different format. This guideline covers permanent vascular access for haemodialysis but does not cover temporary access for haemodialysis or access for peritoneal dialysis, and a number of relevant clinical topics have not been covered, though they would be appropriate to include in future versions, including: access for haemodialysis initiated during pregnancy, and the management of redundant AV access after successful transplantation.

However, the update is broader than previous versions. For example, sections covering access complications have been greatly expanded including those arising with AV access and catheters, and a specific section has been written addressing central venous stenosis (an important but sometimes under-appreciated condition). In many aspects this update seems to make no substantial change to previous guidance (as with the general preference for fistula access, for example, where the literature remains dominated by large observational studies), however whilst key concepts remain valid, their understanding has developed, and the guideline aims to provide greater context, encouraging a more holistic interpretation.

Discussions about haemodialysis vascular access require a number of technical terms, and for the lay reader there is therefore a glossary explaining these for quick reference. Additionally, statistical concepts are important to understanding the rationale, but may be unfamiliar to some readers - these are therefore explained in another appendix, though these explanations are necessarily brief, and standard introductions to statistical analysis should be read by those needing more detail. We have tried to maintain a high standard of readability since conceptual understanding is the key goal, and as the guideline is not intended to replace handbooks, review articles or original papers, it seems correct to favour readability over detail.

## Guideline 1. Access choice considerations

1.1  We recommend focussed access advice for all adults and children anticipating or undergoing a period of haemodialysis, providing simple information outlining the relative merits of a range of access types [1C]
1.2  We recommend treating access choice as a patient decision, supported by the multidisciplinary team, allowing adequate consideration time, taking into account individual patient characteristics and priorities [1C]
1.3  We recommend advising fistula formation for adults and children with suitable anatomy and a likelihood of prolonged haemodialysis [1B]
1.4  We suggest advising catheter access for very small children, and when a short period on haemodialysis is anticipated [2C]

## Rationale

Rather than the technology of membranes, pumps and water purification, the history of dialysis is most closely associated with the development of vascular access. It was not until Belding Scribner’s development of a continuously flowing arterio-venous shunt that long term dialysis became possible, and the exponential growth in dialysis numbers in the last quarter of the 20th century owes as much to two further access inventions, the fistula and the catheter, as it does to medical or political will. Many excellent histories are available [1] but in summary, as the modern era of dialysis was beginning around 1980, shunts had almost disappeared in favour of fistulas, whereas by 1990 prevalent patients were divided between fistulas and catheters, with a smaller number of patients using grafts.

In America in particular, graft use was popular and supported by manufacturers, but their favourable short-term outcome was followed by an increased complication rate and the need for regular intervention. The original motivation behind the ‘fistula first breakthrough initiative’ was to reverse this trend and hence promote fistula access. An unintended consequence of diminished graft use was increased reliance on catheters, and as this became apparent towards the end of the 1990’s, along with the first observational studies of access mortality associations, the mantra of ‘fistula first, catheter last’ was born.

This hierarchical concept of ‘best access’ (in which a fistula is better than a graft, which is better than catheter) became consolidated in literature, widely accepted, and incorporated into guidelines during the first decade of millennium, with financial incentives in a number of countries. The NHS adopted the concept in 2011 with the introduction of a best practice tariff for haemodialysis, which purchases dialysis sessions from institutions according to the access used, with catheter patients attracting 20% less income than those on a fistula or graft. In the most recent registry audit, just under 70% of prevalent haemodialysis patients were using a fistula or graft, with the latter contributing about 4% [2].

### Evidence comparing access types

Studies of access type generally focus on one of three kinds of outcome: mortality, access complications (such as infection or bleeding) and patient experience (including access stability and satisfaction). Access failure may be regarded as an access complication (leading to symptoms and risks arising from delayed dialysis and further access procedures) or as one element of access stability (initial success, maintenance and durability) which impact patient experience (treatment burden and interruption of normal life) more than medical outcome. We appreciate both perspectives but favour the latter view, discussing access stability alongside other aspects of patient experience.

Whilst the statements for vascular access provision in adults and children are similar, the studies and considerations underpinning them are slightly different. Much of what we discuss overall is relevant to children, but we have added also a paediatric section highlighting considerations specific to children, some of which may be relevant to young adults also.

A. ***Mortality***

A large number of studies observe that patients dialysing by fistula have longer survival than those dialysing by catheter. This wealth of data is perhaps best summarised by Ravani’s meta analysis: in 62 cohort studies, comprising half a million patients, higher mortality was seen in patients dialysing with catheters compared to fistulas (RR 1.53, 95%CI 1.41–1.67) and catheters compared to grafts (RR 1.38, 95%CI 1.25–1.52) [3]. Similarly, in 200 studies, Almasri observed 2-year mortality at 15%, 17% and 26% in those dialysing by fistulas, graft and catheters [4]. So a large body of data, systematically summarised, confirms the observation that, even after adjustment for age and other variables associated with catheter use, dialysis by catheter associates with poorer outcomes, implying that catheters are a less safe form of access. The separation in mortality between fistulas and grafts is smaller, with patients dialysing by graft at modestly higher risk than those on fistulas (RR 1.18, 95%CI 1.09–1.27) [3].

Although adjusted for age and known comorbidity, both Ravani and Almasri highlight a high risk of bias due to selection, since catheters and grafts may be favoured when prognosis is poorer. DOPPS studies, recently summarised [5], go some way to addressing this concern, since analysis at facility level (rather than patient level) reduces selection bias. Covering 400 facilities in 20 countries, fistula prevalence was seen to vary from 49% (Canada) to 92% (Russia), with provider preference appearing to influence choice rather than comorbidity. Fistula prevalence remains associated with outcome: facilities in which fewer patients dialyse by fistula had greater mortality (HR 1.14 per 20% greater catheter proportion, 95%CI 1.06–1.22, and HR 1.07 per 20% greater graft proportion, 95%CI 1.01–1.13).

However, in observational studies it is not access as *intended* which associates with outcome, but access *achieved*, which is itself an intermediate outcome. Bias arises not just from selection therefore, but from unmeasured confounders which drive both outcomes (achieved access and mortality). The issue of bias in these studies therefore brings into question the superiority of fistulas in terms of mortality, and at least suggests a smaller causal effect than indicated by the observed association. The debate is not simply a matter of statistical theory, as several recent studies have probed the mortality-access association more deeply, finding clearer evidence for the existence of bias:

1. Some studies suggest catheters continue to be harmful long after removal. For example, in a study of over 17 000 patients receiving a kidney transplant after at least a year of haemodialysis, catheter access (at haemodialysis initiation) was associated with higher post-transplant mortality than a fistula (HR 1.54, 95%CI 1.23–1.89) despite the fact that the catheter would have been long since removed [6]. Effects which are so delayed are implausible, and likely only present due to selection bias at the time of insertion.
2. The mortality disadvantage of catheters appears not to be due to complications. In a study of over 6000 patients, the same catheter-mortality association was seen in those with and without an access complication [7]. Evidence of a plausible mechanism linking catheters with increased mortality is therefore lacking.
3. Fistula attempts which are unsuccessful still appear to confer a mortality advantage. For example, out of 98 000 patients starting dialysis via a catheter, mortality in those with a previous fistula attempt was lower than those with no attempt (HR 0.66, 95%CI 0.64–0.68) despite the attempt being unsuccessful [8]. The beneficial effect of fistula formation therefore extends to those who dialyse via catheter anyway, since their fistula was unsuccessful - this strongly suggests selection bias as the mechanism. A similar effect was found by Quinn, who noted also the paradox that a fistula attempt appears protective against a wide range of infectious and non-infectious causes of death [9].

The evidence base for an access hierarchy based on mortality is therefore insecure, with recent studies highlighting uncertainty. Although a supportive consideration, we feel, along with the 2019 KDOQI guideline authors, that mortality is insufficient as a sole rationale for access advice: ‘There is inadequate evidence for KDOQI to make a recommendation on the type of vascular access preferred in prevalent haemodialysis patients based on vascular access outcomes, patient hospitalizations, or mortality’ [10].

Regardless of its certainty or effect size, any mortality reduction offered by fistula access will be time dependent, with the advantage diminishing in older patients and others with limited prognosis. In a decision analysis using published relative risks (e.g. catheter vs fistula mortality RR = 1.32) the fistula survival benefit (vs catheter) was strongly age dependent. Whereas a 40-year old non-diabetic woman could expect a fistula decision to deliver up to 3 additional years of life, in an 80-year old diabetic woman the lifespan advantage is just 3 weeks [11].

B. ***Complications***

Complications may arise from all types of vascular access, though the nature, severity and frequency of complications varies between access types. The problems of catheter-related infection and venous stenosis are perhaps best documented: for example, in a cohort study of over 1000 incident haemodialysis patients remaining on catheter access, specific complications such as bacteraemia and central venous stenosis occurred during the first year in 9 and 2% respectively [12]. But no access type is complication free, with infection, limb dysfunction, access-related heart failure, stenosis and haemorrhage being the main problem types. It is often difficult to compare the relative importance of complications with frequency of the complication providing only one dimension: severity of the complication and long-term impact on the individual are also relevant, but harder to quantify. We briefly summarise comparative studies by type of access complication:

1. Access infections may be localised to the catheter exit site, tunnel or AV needling site, but the most serious infections are bacteraemic sepsis, and distant haematogenous infections. Bacteraemia in haemodialysis patients is commonly though not exclusively access-related, and rates vary markedly by access type, being highest in those dialysing by graft or catheter. For example, in a 12-month national registry study covering 500 Staphylococcus aureus bacteraemia events in haemodialysis patients, rates in patients dialysing by fistula, graft and catheter were 1.3, 4.7 and 5.7% per year respectively, with 0.4% per year seen in patients on peritoneal dialysis [2]. Although non-access sources contribute more commonly to Gram-negative infections, these too differ by access type: in a single centre study covering 1491 patient-years, Gram-negative bacteraemia was observed in fistula, graft and catheter patients at rates of 4.0, 8.8 and 7.7% per year respectively [13]. Facility experience may be a modifying factor, with catheter infections appearing to be less frequent in facilities with higher catheter prevalence (RR 1.91 comparing lowest to highest catheter prevalence facilities, 95%CI 1.39–2.63) [14].
2. Access-related limb dysfunction is largely (but not exclusively) limited to patients dialysing by fistula or graft, and may be due to circulatory insufficiency (steal syndrome) or neuropathy (due to ischaemia or entrapment), with the former often treatable. Frequencies are dependent on definitions, since steal syndrome is often mild, but cases requiring intervention are not rare, particularly in some groups: in 602 patients undergoing fistula formation aged 55(±13) years, hand ischaemia requiring intervention developed in 26 (4%), with risk factors including female gender (OR 3.17, 95%CI 1.27–7.91), diabetes (OR 13.62, 95%CI 1.81- > 100), and coronary artery disease (OR 2.60, 95%CI 1.03–6.58) [15]. In addition to patient characteristics, steal syndrome appears related to access size/site rather than type, with progressively increasing risk observed in forearm fistulas, grafts, and upper arm fistulas.
3. Access-related heart failure arises from the additional blood flow which accompanies fistula or graft access, which usually increases heart output by at least 15%. Such changes are related to access flow, so that effects are most marked with larger (usually upper arm) fistulas [16] but most haemodynamic effects don’t lead to symptoms. Estimating clinical frequency is difficult because other causes of heart failure are so common, and congestive features of heart failure are controlled by dialysis, so this is perhaps best studied in the pre-dialysis setting. For example, in a prospective study of 562 patients with advanced kidney disease (GFR < 30), followed for median 15 months, episodes of heart failure were identified in 95 patients. Heart failure was unrelated to GFR, but more common in those undergoing fistula formation (29 vs 12%), in whom it was identified after a median (IQR) interval of 7(4–20) weeks. Amongst traditional risk factors for heart failure (age, hypertension, coronary disease), prior fistula formation was the strongest (OR 9.54, 95%CI 4.84–18.81, p < 0.001) [17]. Despite the limited literature, patients whose symptoms were substantially improved by fistula reduction or closure are within the experience of most vascular access clinicians. The pathology is usually multifactorial, suggesting that this is mostly a concern for those whose heart function is already impaired before fistula formation.
4. Stenosis is a complication that affects all access types. Fistulas and grafts may develop stenosis, mainly through the development of neointimal hyperplasia thought to arise from turbulent flow during treatments and repetitive cannulation [18, 19]. Development of stenosis in fistulas and grafts affects flow through the vessel and can progress to access thrombosis [20] so minimising stenosis is important to preserve future fistula/graft function. Central venous stenosis is largely a complication of catheter access, though non-dialysis catheters and pacemakers may also be causative so that fistula and graft patients are not completely spared [21]. Frequency varies by threshold for diagnostic imaging, since the clinical effects are highly variable, ranging from a large asymptomatic group to a smaller number with facial or upper limb swelling, or hypotension. Rather than symptoms the main importance of central venous stenosis is the detrimental effect on subsequent vascular access, with future options more limited and less durable. This complication, aspects of which are covered in more detail in Chapters 4 and 7, is therefore more concerning in younger patients and those with a favourable prognosis.
5. Access haemorrhage takes many forms, from the common fistula ‘blow’ (miscannulation bruise) to dialysis disconnection haemorrhage (for example due to venous needle dislodgement or catheter hub loosening) which is perhaps the most dramatic. Though miscannulation is rarely serious, it is usually painful, and may accompany around 4% of dialysis sessions [22], affecting 89% of patients during the first 6 months of cannulating a new fistula [23]. More threatening perhaps are haemorrhages taking place outside the dialysis unit, for example due to needle site ulceration. Haemorrhage incidents are thought to be rare, though the true incidence is uncertain due to inconsistent reporting, but some studies have provided high quality data on fatalities due to haemorrhage, suggesting occurrence with all access types, but a higher risk in patients dialysing via graft. In a study of 1581 fatalities in dialysis patients coded as ‘haemorrhage of vascular access’ (mostly occurring outside the dialysis unit) authors estimated that access haemorrhage caused 0.4% of all US haemodialysis deaths between 2000 and 2006, with graft access, hypertension and prior access complications all conferring higher risk [24].
6. Air embolism is a rare dialysis complication, due to air entering the circulation, occurring for example with faulty tubing or unclamped ports. It presents rapidly with respiratory or neurological symptoms, sometimes leading to cardiac arrest, and carries a high mortality, with persistent disability common amongst survivors [25]. Although rare the true frequency is unknown, with literature largely confined to case reports and safety incidents. The risk of air embolism is greater with catheters, where pressure is often negative (therefore sucking air in rather than bleeding), than with fistulas and grafts, where pressure is usually higher, (though it can still occur). When access-related, it may occur at the time of catheter insertion or removal, or at any time due to catheter unclamping or misuse (eg. by a cognitively-impaired patient or non-dialysis-trained clinician) or faulty tubing.

Apart from infection and miscannulation, these complications of access are uncommon, though sometimes serious, and covered in more detail in other chapters. The distribution varies greatly by access type, and some are specific to a single type, but both risk and impact are also highly dependent on patient characteristics. There is therefore no such thing as the average patient, though fistulas consistently emerge as the least liable to adverse effects or hospitalisation, whereas the difference between grafts and catheters is less clear: graft complications appear similarly frequent, though catheter complications may be more serious.

C. ***Patient experience and treatment burden***

Whilst patients’ experiences of vascular access are less well studied than other outcomes, they are equally important, and there is a gradually increasing body of literature in this area. Experience depends partly on clinical aspects (symptoms and defined complications) but also on treatment burden (which depends on access stability) and patient-specific priorities/treatment goals, and is therefore highly subjective. We briefly summarise comparative aspects of access stability and overall patient satisfaction.

1. Initial access functionality is around 98% for catheters, whereas around a quarter of fistulas are unsuccessful initially, increasing to around a third when including those which are abandoned early. The best fistula outcome estimates come from a meta-analysis of 62 cohorts covering over 12 000 fistula formations: 77% were successfully used for dialysis initially, but by 2 years the number still working was down to 64% [26]. In a Scottish study including all nine kidney centres, 30% of fistulas never worked, increasing to 34% during 12 months’ follow-up [27]. Patient characteristics such as older age, cardiovascular disease and prior fistula failure are consistently associated with poorer fistula success rates [28], but these associations are too weak to reliably predict outcome for individuals. Fistula success or otherwise is only determined at dialysis initiation, and not all fistulas are ever required. In older patients in particular, kidney failure progresses slowly and patients may reach the end of their lives for other reasons, without requiring dialysis. In a study of 2741 patients over 70 undergoing pre-dialysis fistula formation and then followed for 2 years, only two-thirds actually needed their access: 14% died and 20% remained well, without ever requiring dialysis [29]. Similarly in an observational cohort study in Scotland, after a mean follow-up of 12 months, 29% of fistulas (166/582) were not in use for haemodialysis [27]. Pre-dialysis fistula formation therefore creates treatment burden without benefit for a significant number of (mostly older) patients. Catheter function is immediate, and placement is therefore usually concurrent with dialysis initiation, so this problem doesn’t arise. Functionality with modern grafts can be achieved more reliably and quicker, so they allow a delayed access plan closer to dialysis initiation.
2. Once functional, access durability also varies between access types, with fistulas generally lasting longer. For example, in 200 studies covering 800 000 patients, Almasri found primary (without maintenance) patency (95%CI) rates with fistulas, grafts and catheters to be 55(52–58)%, 40(35–44)% and 50(41–61)% at 2 years [4]. Secondary (with maintenance, therefore total functional time) patency for fistulas and grafts was 63(59–67)% and 60(55–65)% at 2 years. Maintenance usually involves surgery or interventional radiology, with additional treatment burden therefore, in particular with grafts. Patency figures in modern studies include initially unsuccessful access, so these rates equate to the loss of initially successful fistulas at around 10% per year, and catheters/grafts at around 25% per year. The improved initial functionality of grafts is therefore offset by higher maintenance and shorter total durability. This outcome is particularly important, with the SONG-HD study (Standardised Outcomes in Nephrology) identifying vascular access function as the most important outcome for both patients and healthcare professionals [30].
3. Patient satisfaction with their vascular access has been compared in two studies, both favouring fistula access. In a Canadian study including two cohorts of 132 and 140 patients, using a validated questionnaire, satisfaction scores in patients dialysing by fistula, graft and catheter were 6.5, 5.2 and 5.9 (with higher scores indicating greater satisfaction) [31]. And in a study of 749 patients from Birmingham, using a similar validated questionnaire (but in which lower scores indicate fewer patient-perceived problems) Field found scores of 5.1, 7.2, and 6.6 in patients dialysing by fistula, graft and catheter respectively (p = 0.004) [32]. Differences between these satisfaction scores were explained by specific patient-perceived problems, such as pain (perhaps more common with AV access, p = 0.068), bleeding and bruising (distinctly more common with AV access, p < 0.001), redness and infection (more common with catheters, p < 0.001), and clotting (more common with grafts and catheters, p = 0.008). Perhaps surprisingly, daily physical symptoms were generally of more concern to patients than delayed departure from dialysis or hospitalisation [31]. Overall quality of life has also been linked to access, with Nimmo finding that AV access was associated with reduced disease burden and improved physical and mental composite scores using the KDQOL questionnaire in 738 patients in Scotland [33]. Though much like mortality data, this study would be biased by any association between quality of life and access selection, which is quite likely.

Qualitative research also demonstrates a significant burden associated with vascular access regardless of type, best summarised in Casey’s thematic analysis of 46 studies including 1,034 patients [34]. Their synthesis demonstrates that vascular access for patients is not just about having a fistula, graft or catheter for dialysis sessions, but acts as a constant link to a life sustaining treatment, creating anxiety and feelings of vulnerability. Vascular access can cause patients concern with physical intrusion, fear of cannulation, a threat of complications and failure, dependency, disfigurement, impingement on their life including family life and a constant reminder of their need for haemodialysis [34]. However, it also is associated with self-preservation, enabling them to have haemodialysis. It is easy to see therefore how important and highly personal these decisions are, since they affect patients deeply, going far beyond clinical outcome.

D. ***Evidence summary***

If one were to generalise access outcomes (for a moment treating patients as a single group) then a wealth of literature associates achieved access with mortality, consistently suggesting quite a large effect, favouring fistulas followed by grafts, with catheters last. However, all of this type of literature shares the same statistical bias, and causal effects are therefore unclear. General fistula preference is more firmly supported by studies of complications and patient experience. Although complications occur with all access types, their distribution favours fistulas as the least harmful, with no clear distinction between grafts and catheters. And patients are generally most satisfied with a functioning fistula, with grafts proving to be the most problematic from their perspective.

However, many access considerations are highly individual. Mortality advantages in particular diminish with age and comorbidity: for patients with limited time, longevity is not a major consideration, with greater priority given to the present moment, and convenience rather than safety [35]. Some complications are also more relevant to specific groups, such as central venous stenosis, which becomes less of a concern as prognosis shortens. Satisfaction depends very much on patient priorities, with treatment burden in particular resented by those whose time is limited. And for many patients, access effects are highly personal, going far beyond clinical outcome.

### From evidence to decision making

The concept of ‘best access’ informs standard clinician advice, but it is also an oversimplification which ignores knowledge uncertainties, patient variety and choice. This knowledge gap is increasingly recognised by clinicians: in a survey, 86% of Canadian and 66% of European nephrologists indicated their willing to participate in a randomised trial of access type in incident patients at high risk of fistula failure [36]. Another group has initiated a feasibility study, in which patients over 65 who started dialysis via catheter are randomised to fistula formation or a long-term catheter strategy [37].

In view of the knowledge gap, the stance of many clinicians and authors seems overconfident, and perhaps occasionally paternalistic, often discussing ‘educating our patients’ with insufficient recognition of uncertainty or appreciation of the individual perspective [38]. In some guidelines on vascular access, there is no mention of patient views or their involvement in decisions [39].

Perhaps unsurprisingly many patients decline clinician advice, and whilst some of this stems from misunderstanding (‘my catheter works so what’s the problem?’) much is rational [35]. Patients rarely make decisions about vascular access the same way healthcare professionals do, placing less emphasis on clinical outcomes and more on practical effects on their day-to-day lives [40, 41]. Information is therefore needed as much as advice and providing clear information has been shown to engender trust [42, 43], improving acceptance and retention of the information provided. Cavanaugh assessed haemodialysis patients’ knowledge and compared this to access type, demonstrating an association between haemodialysis knowledge and dialysis by fistula or graft (p = 0.05) [44].

But providing real knowledge to patients should not be seen as a tool to promote particular choices, but an essential step in ensuring choices are informed by understanding, as well as consistent with personal circumstance. This may be particularly important pre-dialysis, as once patients start haemodialysis they are more likely to choose the ‘status quo’ over their true optimal access [44]. How to achieve this is more of a challenge: thinking about the ‘right access, right patient, right time’ is more common, and KDIGO advocates the use of a patient-specific ‘life plan’, though few details are provided.

One concept which may have outlived its utility, however, is the idea of numerical targets and the associated incentives for institutions based on access type achieved. Although appropriately intentioned to reduce system barriers to AV access achievement, targets and incentives are dependent on the concept of universal ‘best access’, and may work counter to patient choice. As our understanding of access evolves, and the concept of best access is replaced by one of ‘right access’, it seems clear that patient decisions should be free from external considerations which might bias clinician advice. The belief that patients should be at the centre of access decision making is inconsistent with the idea of an institutional target, or an incentive which rewards institutions when a particular decision is made. Process targets (such as wait time for access procedures) should perhaps be considered instead, since they don’t impact choice and would therefore be more supportive of patient-centred care.

### Paediatric considerations

Children with end-stage kidney disease have a lifetime of kidney replacement therapy ahead of them. Whilst either pre-emptive transplantation or peritoneal dialysis is the initial modality in many, with less than half starting on haemodialysis, over their lifetime almost all such children will experience haemodialysis. A long term view of dialysis options is therefore necessary from the start, including vascular access use and venous preservation. Although transplantation is the optimal modality and available for many at an early stage, a quarter of children experience transplant failure and return to dialysis, even before moving to adult programs [45, 46].

As with vascular access in adults, fistulas have several advantages over catheters in children, though with different and sometimes greater emphasis. In particular, central venous stenosis compromises future options for AV access and makes catheter insertions more difficult (Chapter 7). Catheters are the principle cause, and once acquired it is usually permanent or recurrent after treatment, and therefore of particular relevance to those facing many years of kidney replacement therapy [47, 48]. This complication is a particular concern for children therefore, pertinent not just to the current episode of haemodialysis, but to vascular access for perhaps multiple periods of haemodialysis in their future life.

Similar to adults, the risk of infection is greater with catheters than with fistulas in children. In a retrospective UK study of access outcomes in children on haemodialysis for at least a year, comparing fistulas (N = 20) with catheters (N = 5), fistulas were associated with lower rates of infection (3% v 38% bacteraemia episodes per year, p = 0.002) and access-related hospitalisation (0.4% v 3.1% per year, p = 0.004) [45]. This finding is confirmed in large registries: in an International Paediatric Haemodialysis Network (IPHN) study, which included 552 children over 314 patient-years, the catheter-related infection rate was 46% per year, requiring access replacement in 47% of cases, whereas infections were not observed in children with fistulas [49].

As in adults, fistulas in children are more durable than catheters. In the IPHN registry study [49] access dysfunction requiring intervention occurred more often with catheters (every 18 months) compared to fistulas (every 28 months). And in a large retrospective study covering 182 catheter insertions and 107 fistula formations, catheter failure occurred much earlier than fistula failure, at 0.6 years (95%CI 0.2–1.0) versus 3.1 years (95%CI 1.2–5.1). At all time-points up to 4 years from access formation, a greater proportion of fistulas than catheters remained functional (p < 0.001). Regardless of access type, younger age appears to increase the risk of access failure [50]. In some studies higher dialysis adequacy is seen in children dialysing via fistulas compared to catheters [45, 51], and in children a narrower gauge of catheter is typically used than in adults. The experience of living with a catheter is also different for children, with the ability to swim being important for many.

Vessel size sometimes limits fistula options, particularly in younger children, but only a few studies report details of fistula assessment and outcome. In a single centre study, assessment and outcome were reported in 12 children undergoing fistula formation, with median(IQR) age 9(6–14) years and median(IQR) weight 27(14–67)kg [51]. Median(IQR) artery and vein diameters pre-operatively were 2.7(2.0–5.3)mm and 3.0(2.0–5.0)mm. All fistulas matured though two required angioplasty to achieve it, and one only reached maturation after a year. One child was transplanted before maturation so the fistula was not required, and in two children needling was delayed by the need for extensive psychological preparation. Though children’s vessels are smaller in size, they are usually better in quality, with less calcification for example. But as with adults, formation is less commonly attempted with smaller vessel diameters, and although occasionally reported using microvascular surgical techniques [52], fistula formation is most unusual in children younger than 3 years, or weighing under 10 kg. Grafts are rarely used in children, accounting for less than 2% of access [49, 53].

As with adults, non-anatomic considerations are important in children. Catheters are frequently life saving when kidney failure presents rapidly or at an advanced stage, and are also favourable for short dialysis periods: in many children haemodialysis is required for only a few months as a bridge to live-donor transplantation, and the average waiting time for a deceased-donor transplant is one year (compared to three years in adults). Some children have complex conditions which limit transplant options, and longer wait times can to some extent be predicted: we therefore suggest that fistula formation should be considered for children for whom transplantation is unlikely within 6 months.

The evidence base is limited by the relative rarity of end-stage kidney disease in children, and as with adults a reliance on observational data, associated with the same types of bias (principally patient selection and as-treated analysis). However, a consensus exists over the benefit of fistulas over catheters in many instances, which may exceed that in adults, due to the longer life expectancy of children beginning kidney replacement therapy. Whilst this section discusses children and adults separately, there is no abrupt transition in the principles of treatment, and decision making in younger adults may resemble that of children more than older adults. One constant is that decisions about access type used for haemodialysis are highly personal, requiring multidisciplinary consideration of individual circumstances and preference.

In ‘real world’ studies, though the potential advantages of fistulas are acknowledged, catheters remain the main type of access in children: in 2019 the IPHN registry reported 26% of children prevalent on haemodialysis using fistulas, despite a median age of 12, and only 5% of the population being under 2 years [49]. A reluctance to consider fistulas for children may arise from limited expertise or experience in all aspects of access care, including fistula formation, fistula cannulation, needling anxiety and managing fistula complications. Infrastructure to support vascular access provision in children needs to be developed, to enable appropriate children to benefit from fistula use for haemodialysis. A dedicated paediatric vascular access clinic can provide a focal point for education, assessment and ongoing management of vascular access in children [46, 54, 55].

### Conclusions

Whilst summarising the evidence base we have deliberately highlighted its uncertainties, to allow a balanced dialogue acknowledging reasonable patient concerns, and allowing ‘fistula advantage’ to be interpreted within the context of clinical status and patient-specific goals of treatment. There are two main conclusions which can be drawn:

1. It is logical to routinely favour fistula access in order to achieve minimal complications and maximal patient satisfaction, and this may also improve clinical outcomes. The same logic does not generalise confidently to patients at high risk of fistula failure, or those expecting a limited dialysis prognosis, so that there is no universal ‘best access’. The reasons for routinely favouring grafts over catheters are less clear, since satisfaction and complication rates are more comparable, though they are a reasonable choice.
2. The access decision always depends on the individual patient’s values, and is a *choice*. Patient decisions should be facilitated by information and advice, but protected from provider preference, and supported with multidisciplinary input. Considerations are highly individual, access experiences are highly personal, and patients need to be placed at the centre of the decision process. Numeric institutional targets for fistula prevalence are inconsistent with an individualised choice-based approach.

## Guideline 2. Access preparation, assessment and timing

2.1  Whilst optimal vascular access timing depends on patient and institutional factors, we suggest access referral, and if suitable fistula formation, are appropriate for any adult or child planning haemodialysis and likely to start within 12 months, whereas vascular access education is appropriate at any stage of kidney disease [2C]
2.2  We suggest that all adults and children likely to require long term haemodialysis, and their carers where appropriate, should receive education on vascular access and vein preservation, which should be tailored to their individual situation, and may be delivered by various members of the multidisciplinary team [2C]
2.3  We suggest advising and facilitating avoidance of cannula insertion, and where possible all vessel puncture, proximal to the wrist in the non-dominant or fistula-planned arm for adults where there is a high lifetime risk of kidney failure, and bilaterally in children [2D]
2.4  We suggest that a patient’s decision (adult or child) on whether and where to proceed with AV access formation is best informed by combined clinical and ultrasound assessment [2C]
2.5  We suggest central vein imaging prior to AV access formation, with either conventional or cross-sectional venography, in adults and children with clinical features or high risk of central venous stenosis [2C]

## Rationale

Adequate time is required for the selection, formation, and maturation of dialysis access so that it is available when needed for dialysis initiation. In particular, when fistula access is planned, it needs to be ready for cannulation when dialysis is required: fistula establishment may be undermined either through failure of primary patency (never developing) [1], or by insufficient maturation (needing more time to develop), and an average maturation period of 10 weeks should be expected before a fistula matures to the point of sustainable use [2]. Grafts do not need to mature, though depending on the type of graft a period of one or two weeks may be required before cannulation begins, so that it is incorporated into the tissue and doesn’t bleed after dialysis. More modern ‘early cannulation’ graft technology has allowed increasing use of grafts that are self-sealing and can be cannulated within hours of surgery [3].

Determining when to pursue vascular access therefore requires anticipation of when it will be required. This can be challenging, as the GFR at dialysis initiation is variable, and the timing of dialysis initiation even more so, being influenced by a number of factors including the rate of GFR decline (which may be non-linear), age, comorbidity, proteinuria and the impact of intercurrent illness [4]. The duration of specialist care before dialysis is therefore important, and was the subject of a Cochrane systematic review summarising 40 studies comprising over 60 000 patients starting dialysis, separated into early (over 6 months prior to dialysis) versus late nephrology referral [5]. Early referral resulted in reduced temporary access (OR 0.47, 95%CI 0.45–0.50) and reduced mortality after dialysis initiation (OR 0.69, 95%CI 0.62–0.69). These benefits appeared to be independent of comorbidity (such as diabetes or vascular disease) and GFR, though since all studies were observational, they may be have been biased by referral patterns (e.g. referrals deferred due to intercurrent illness). It therefore appears that better access preparation could explain improved outcome in earlier referred patients, implying that access referral less than 6 months before dialysis, is often too late.

However, too early a referral may expose patients to creation and maintenance of an access that may never be required, due to competing events, such as transplantation, or death before kidney failure develops. These issues are particularly relevant in older people, in whom competing illness is more common, and fistula outcomes less favourable. In a large American study, Hod reported outcomes in 17 511 patients over 67 who started dialysis after prior fistula formation: 45% used a graft or catheter for dialysis initiation, rather than the fistula as planned [6]. Looking at the timing of fistula formation, successful fistula dialysis was less likely with formation only 1–3 months before dialysis (OR 0.49, 95%CI 0.44–0.53) or 3–6 months before (OR 0.93, 95%CI 0.85–1.02), but formation over a year before was no better than 6–12 months before starting dialysis. There is therefore an optimal window for access referral and formation, that is around 6–12 months before dialysis initiation, though predicting the latter event is difficult. This window may also be affected by institutional factors such as the expected time waiting for surgery, or for procedures to assist maturation. These issues are well summarised in a review article by Woo [4].

Where the window of opportunity begins is likely to vary, and therefore using a single GFR threshold for vascular access planning may not be appropriate for all. A range of GFR values by which services may wish to consider starting vascular access planning may account for the variation to a better degree, yet should only be used as a guide. Few studies have assessed this, but in a simulation study based on published outcomes and rates of kidney disease progression, Shechter modelled different strategies aiming to maximise fistula dialysis and minimise unnecessary fistula formation, supporting an optimum GFR range of 15–20 ml/min for access referral [7]. The kidney failure risk equation (KFRE) has been used to determine a threshold level of risk that would facilitate optimal selection of patients for placement of dialysis access. A KFRE-based threshold of 20% annual risk (>40% over 2 years) has been described as superior to GFR-based thresholds in generating the highest number of optimal dialysis starts with a mature access in observational work [8]. GFR threshold strategies have the advantage of easier implementation because they do not require forecasting dialysis initiation. In contrast, time window strategies may be more accurate since they consider individual characteristics and the rate of kidney disease progression, but they are harder to apply in practice.

In children, sufficient time is necessary for psychological preparation as well as pre-operative investigation, fistula maturation, and any further intervention for non-maturation. Whilst paediatric registries report a median(IQR) interval of 62(37–134) days between AV access formation and cannulation, independent of age [9, 10], angioplasty to assist maturation is required in 17–28% of fistulas in children [9, 11–14], and with time allowed for psychological preparation the overall process from pre-operative assessment until the fistula is functional (regardless of fistula location) requires an average of 6 months [9, 15, 16]. Access referral is usually considered when GFR is below 30 ml/min/1.73 m^2^ (estimated by Schwartz formula [17]), or otherwise when haemodialysis is expected within 6–12 months [18].

Education however has no time window limitation, and counselling patients about the main risks and benefits of each access type is widely regarded as worthwhile, as explored and summarised by Moist [19]. Improved patient understanding allows more informed decision making, and facilitates delivery of a more personalised vascular access strategy [20], and observational data suggest that access education programs are associated with increased AV access at dialysis initiation [21, 22]. A structured approach to education should be encouraged, that focuses on simple concepts, reflecting on individual circumstances and goals, and may be delivered through a variety of different methods, such as face to face, group education, or written literature.

Monitoring kidney disease requires frequent blood sampling, exposing patients to a large cumulative number of vein punctures, many of which occur outside the nephrology clinic, in primary care or other specialty services. Vessel puncture for blood tests, and cannula insertion in particular, are widely regarded as a cause of vein scarring and stenosis, which may limit the number of sites suitable for fistula formation, and reduce vein quality compromising fistula success. There is, however, little high quality data that explores or quantifies these risks, nor is there data on patient experience of blood sampling from more peripheral sites such as the back of the hand. Nonetheless most clinicians consider it important to advise patients about vein preservation once the need for dialysis becomes likely, so that they can avoid vessel puncture in potential fistula locations, within what seems practical and acceptable to the individual. Fistula planning typically favours the non-dominant arm (to limit the impact of rare neurovascular complications, and allow use of the dominant arm during dialysis) and distal locations first (to preserve more access options for the future). Vein preservation is therefore often advised proximal to the wrist of the non-dominant arm, to preserve the forearm cephalic, antecubital and upper arm veins, whilst arterial punctures on the non-dominant arm should also be avoided where possible.

Thorough preoperative assessment is the cornerstone of vascular access planning, considering anatomy and the probability of success, to inform the decision on whether and where to proceed. History should include heart disease or devices, and prior central venous access. Examination should assess arterial inflow and venous outflow, considering vessel size, depth, flow pattern, degree of calcification, and if there is a suitably straight section available for cannulation.

Ultrasound, though not universally used, enhances this assessment. It is probably more objective than clinical examination, with excellent inter-observer agreement for typical vessel measurements [23], but whether routine ultrasound use improves clinical outcomes is uncertain. One systematic review focussing on four studies comprising 450 patients found no advantage with pre-operative vessel imaging over clinical assessment alone [24]. But in another review covering 402 patients, including two of the same studies, Wong reported improved fistula success with ultrasound planning, though the difference may have been due to chance (81% v 69%, p = 0.11) [25]. It seems likely that there are simple cases, where ultrasound adds little, as well as other cases (including basilic transposition fistulas) where ultrasound is essential, and Smith’s randomised study of selective ultrasound use, which was as good as routine use, seems to support this view [26]. However, diameter thresholds are increasingly advocated for decision making, so it seems that routine ultrasound at least facilitates a patient-centred decision process. In children, a structured approach including history, physical examination and imaging is suggested, similar to adults, though since vessels are typically smaller, diameter measurement by ultrasound is considered essential.

Another imaging consideration is the possibility of central venous stenosis, which may be found in up to 40% of adult haemodialysis patients, and may limit fistula success by impairing venous outflow from the ipsilateral limb [2, 27]. Prior catheter access for dialysis, both the number and total duration, is the dominant risk factor in this group [28, 29], though pacemakers are another important cause. Peripherally inserted central catheters (‘PICC lines’) also appear linked with fistula failure, with the association persisting after adjustment for confounders, including gender, vessel sizes and dialysis catheter history [30]. We suggest that in all patients (adults and children) with kidney impairment needing acute or chronic central venous access, PICC lines, pacemakers or implantable electronic devices, due consideration should be given to the potential impact this may have on their future vascular access options, with central veins protected where possible. Imaging to exclude central vein stenosis should be considered in all patients (adults and children) undergoing AV access creation in the upper limb where there are clinical features suggestive of central venous stenosis, or where there has been previous central venous catheter. Similarly, multiple previous access failures should prompt consideration of the possibility of central venous stenosis. Ultrasound has low sensitivity for diagnosis, and venography (either conventional or cross-sectional) is usually needed.

## Guideline 3. AV access formation and care

3.1  We recommend a multi-disciplinary shared decision, on AV access formation and location, taking into account anatomy, haemodialysis duration and patient preference [1B]
3.2  We recommend routinely favouring distal locations initially for access formation, where supported by vessel anatomy and patient preference [1B]
3.3  We recommend counselling patients to expect poorer outcome if planning fistula formation with one or both vessels less than 2.0 mm diameter [1C]
3.4  We recommend favouring fistula formation over graft insertion in adults and teenage children, except where early cannulation is necessary or anatomy at conventional locations is unfavourable, when a graft may be considered in adults [1C]
3.5  We suggest in adults routinely favouring local or regional anaesthesia, and in children general anaesthesia, to which regional anaesthesia may be added, for fistula formation [2B]
3.6  We recommend that surgical expertise in vascular access creation needs to be established and maintained to achieve optimal clinical outcomes [1C]
3.7  We recommend regular monitoring of new fistulas for maturation, using a ‘look, feel and listen’ approach, supported where necessary by ultrasound [1C]
3.8  We suggest avoidance of low blood pressure peri-operatively and during the maturation period, with review of medications and target weight [2C]
3.9  We recommend in adults an initial assessment to determine maturity for cannulation between 2 and 6 weeks after formation, with investigation arranged for non-maturity persisting beyond 6 weeks. Longer intervals may be more appropriate in children [1C]
3.10  We suggest that the decision to initiate cannulation should follow individualised assessment of the fistula, balancing avoidance of miscannulation with the requirement for prompt access for haemodialysis [2C]
3.11  We suggest adequate preparation prior to initiation of needling in all patients, anticipating the requirement for extensive support in children [2D]
3.12  We recommend an access assessment before every cannulation, using a ‘look, feel and listen’ approach performed by an appropriately trained cannulator [1C]
3.13  We suggest patients who self-cannulate assess their access before every cannulation, using a ‘look, feel and listen’ approach, within the limits of their abilities and with understanding of potential problems [2D]
3.14  We recommend rope ladder or buttonhole cannulation for fistulas, and rope ladder cannulation for grafts, in preference to area puncture wherever possible [1C]
3.15  We recommend unit policies to measure and minimise cannulation complications, which may include ultrasound assisted cannulation or single needle haemodialysis for new or difficult AV access [1C]
3.16  We recommend high quality cannulation training, giving staff time to develop their skill through supervised practice, supported by theory teaching and competency assessment, before performing cannulation unsupervised [1D]

## Rationale

Long-term vascular access for haemodialysis can be provided by a venous catheter or AV access: creation of a fistula or placement of a graft. Ideally AV access should be easy to cannulate, minimally symptomatic, and durable with minimal intervention. Formation and care of high quality AV access remains a significant challenge within the kidney community, requiring complex multidisciplinary collaboration, in particular between experienced nurses, surgeons and nephrologists.

It is widely agreed that where it can be achieved, a fistula is the optimal form of vascular access for haemodialysis, providing the most durable function with the lowest risk of harm. However, no form of access is without drawbacks: for fistulas the long term problem is to achieve reliable cannnulation which maintains fistula function, enables dialysis and minimises complications, whereas the short term problems are maturation time (around 6 weeks after formation when the fistula is developing and not yet ready for use) and primary failure (unsuccessful formation with the fistula never providing reliable dialysis access).

Data on primary failure are difficult to interpret and often affected by the healthcare system, but in a meta-analysis of over 300 studies, Bylsma found that by one year after formation, 64% of fistulas were functioning without assistance, rising to 79% with the use of procedures to maintain or improve the fistula [1]. Within the UK recent studies estimate primary failure rates of 30% [2] and 27% [3] with the fistula either never used for haemodialysis or failing within the first 90 days of use. Early fistula failure leads to further treatment burden and increases the likelihood of patients declining procedures and defaulting to catheter access [4, 5].

Once established and in regular use, AV access needs to continue providing reliable cannulation to enable use for haemodialysis, as this is the sole purpose of access creation. Whilst perhaps obvious, it is crucial to remember that the steps of choosing AV access, selecting location, access formation, and the assessment and management of maturation, all aim to achieve easy and reliable cannulation at each dialysis session over a prolonged period of time. Maintaining this involves high quality nursing and timely management of complications which may occur (some are discussed further in chapter 4) [6]. Complications and the interventions required to deal with them may be burdensome for patients: Stoumpos reported an average intervention rate of 0.48 per patient year [2], and for some patients the experience of living with a fistula and undergoing regular cannulation may be poor, involving anxiety and pain, as well as impacting on body image and quality of life [7–11].

Maximising the success and durability of access function, whist minimising complications and negative experience are all crucial to the welfare of haemodialysis patients. Ensuring high quality delivery of all aspects of access care are therefore important, including location selection, surgery, maturation period, and most importantly the cannulation and routine care of established AV access.

### AV access location

The majority of fistulas are formed in one of three conventional locations, named according to the vessels from which they are formed: radio-cephalic (forearm), brachio-cephalic (upper arm), and brachio-basilic (upper arm using a deeper vein). A number of factors are relevant to the choice between locations. How the fistula may affect the patient’s life both on and off dialysis are important to consider: fistulas in the dominant arm may be more limiting in terms of activities on and off dialysis and, whilst rare, the formation process may damage the structure or nerves of the limb, limiting future activities. Therefore, decisions about fistula location need to consider the patients’ personal priorities for their life on dialysis, and aim to minimise restriction on activities that are important to them, usually favouring the non-dominant arm. Research evidence to support this approach is absent, and one study exploring patient experience found no difference [12], but this can be discussed with patients who can of course choose which arm is assessed first.

A distal (forearm) first approach has traditionally been advocated, in order to preserve more location options for the future, since distal locations are often compromised once proximal (upper arm) locations have failed. Some clinicians favour the very distal ‘snuffbox’ location, which is similarly successful in experienced hands. However, primary failure is more common with distal locations, in children as well as adults [13], in part due to the typically smaller vessel size, though routine distal preference may also be a factor. In a meta-analysis Almasri found improved outcomes with upper arm fistulas including longer secondary patency (HR 0.49, 95%CI 0.28–0.85) than forearm fistulas [14]. An analysis of national data from Scotland found similar results, with upper arm location being an independent predictor of secondary fistula patency (HR 0.48, 95%CI 0.36–0.65) [2]. It is important to minimise primary failure which is currently a large problem, therefore, whilst a distal first approach may have benefits, clinicians should consider for each individual patient whether distal sites will truly lead to a fistula that provides longevity of access for haemodialysis.

Vessel quality may vary and is also important in selecting location, in particular vessel diameter but also including depth, tortuosity and calcification. One meta analysis of 12 studies suggests 2.0 mm as the minimum diameter for optimal success in radio-cephalic fistula maturation [15]. Studies do not support size thresholds however, instead tending to show a continuous deterioration in outcome with reducing diameter: for example, in 116 fistula formations, 80% of which were successful, Malovrh found smaller pre-operative arterial diameters in those which failed (1.6 v 2.6 mm) [16]. And thresholds are also not helpful to a patient with limited options, whose vessels may all be suboptimal. But vessel sizes do give an indication of the outcomes to expect: ‘normal’ rates of success, similar to those reported in studies, can be expected when vessel sizes are typical for those studies, in which artery and vein diameters under 2.0 mm are rare. Patients with suboptimal vessels should be aware of this issue, so that it is considered in their access and location choice. Studies in children typically include smaller vessels with successful formations are described with smaller veins than typically attempted in adults. Where stated in the larger paediatric reviews, veins with internal diameters in the range 1.5–2.5 mm are not unusual.

Consideration also needs to be given to lifestyle issues such as occupation, self-cannulation and appearance. Qualitative research highlights patients’ frequent concern over the appearance of their access, with some keen that it should be easy to cover up [8, 17–19], and some avoiding fistula formation altogether [5]. Rather than indicating the optimum location therefore, these studies emphasise the personal nature of the decision, with clinicians increasingly moving away from universal considerations to an individualised and more thoughtful, patient-centred approach.

The majority of fistulas are either radio-cephalic or brachio-cephalic, both formed using the cephalic vein which runs close to the surface, in the forearm and upper arm. If these two locations are unavailable, due to poor vessel quality or prior use, then a brachio-basilic fistula can be formed, using the basilic vein, which runs more deeply in the upper arm. The depth and closeness to other structures means that the basilic vein often has to be transposed (moved) closer to the surface, to enable easy cannulation for dialysis. This involves a larger operation, often carried out in two stages, separated by a few weeks. Stoumpos compared fistula types, noting lower patency rates in brachio-basilic compared with brachio-cephalic fistulas [2]. However, other studies found outcomes as good as simpler fistulas: in a meta-analysis of 1250 basilic vein fistula formations across 15 studies, 1-year primary patency was 55% (95%CI 47–63%) and secondary patency 75% (95%CI 67–82%), similar to fistulas at other conventional locations [1]. Whilst patient experience with basilic vein fistulas is broadly similar to other types, one study reported greater anxiety over the fistula’s durability [12].

These uncertainties with brachio-basilic fistulas have led some to suggest that graft insertion may be a more favourable option. However, although basilic vein fistulas are more complex to form, they appear to outperform grafts in function: in a meta-analysis of 1509 access formations in 11 studies, Lazarides compared basilic vein fistulas with grafts, observing no clear difference in secondary failure (OR 0.88, 95%CI 0.69–1.12) but a much greater rate of interventions with grafts (1.32 versus 0.54 per patient per year) [20]. The general superiority of fistulas appears therefore to extend to basilic vein transposition. When even the basilic vein is inadequate, it may be possible to form a fistula using the deep brachial vein or even the venae commitantes that run alongside the brachial artery. These veins can be superficialised in a similar manner to the basilic vein, however reported outcomes are less favourable, with increased post-operative complications and shorter patency [21].

### Grafts and thigh access

When vessels for conventional fistula formation have been utilised or are not suitable, graft insertion may be appropriate and should be considered. Since the graft itself is the conduit, no vein is required for needling, though successful graft placement is still dependent on a good calibre artery and vein, for the inflow and outflow anastomoses. The decision regarding configuration is driven by several factors, of which the most important is the size of outflow vein, which should in most circumstances be at least 3 mm in diameter. Other factors include patient age, anaesthetic fitness and obesity. Forearm loop grafts are a useful option for obese patients, in whom deeper upper arm veins may be more challenging to cannulate, and for patients requiring immediate access, since the upper arm is then preserved for future fistula formation. The commonest configurations are an upper arm straight brachio-axillary graft, and a forearm loop brachio-basilic graft: in an observational study of 508 patients comparing these configurations, no outcome difference was seen, though this American study in which initial access was a graft in 90% of patients, may not generalise to UK practice [22].

Compared to fistulas, grafts are less favourable in terms of complications, patient experience, and durability in particular. One year primary and secondary graft patency varies between 40–50% and 70–90% respectively in a range of studies [23–25]. In a review of over 200 studies, 2-year primary and secondary patency rates for fistulas were 55% (95%CI 52–58%) and 63% (95%CI 59–67%). Graft outcomes were inferior with primary patency 40% (95%CI 35–44%) and secondary (procedurally supported) patency 60% (95%CI 55–65%) highlighting the increased treatment burden [14]. Infection rates over the 2 year period were also higher at 13% with grafts (95%CI 10–17%) verses only 2% with fistulas (95%CI 1–4%). However, the superiority of fistulas over grafts is only relevant in those patients who have adequate vessels for fistulas formation. In the absence of suitable vessels, graft placement maybe preferable to a high risk fistula which is likely to fail despite multiple interventions.

In addition to simpler anatomic requirements, primary failure is uncommon, and grafts do not need to mature. A short period of incorporation into the tissues is needed, but grafts can usually be needled from around 2 to 4 weeks - this early reliability allows a delayed access decision, close to the time of starting dialysis. Multilaminar grafts which incorporate into the tissue more quickly have also been developed, allowing earlier needling, usually within 24 hours of placement. These ‘early cannulation’ grafts can be used as emergency access for unplanned kidney failure or for fistula salvage, and they may also be useful when delayed maturation is anticipated and a bridging catheter might otherwise be required: in a meta-analysis of 19 studies 66% of fistula formations (95%CI 57–75%) were accompanied by a bridging catheter [1]. Outcomes with early cannulation grafts are similar to other graft types, with 1-year secondary patency ranging from 41% (N = 37) [26] to 84% (N = 141) [24], with no apparent increase in infection rates (6% over one year).

In attempting to improve patency outcomes and reduce intervention rates, some manufacturers have introduced graft modifications including heparin bonding or carbon lining, neither of which appears to improve outcome [27]. Alterations to the geometry of the outflow end such as expanded or spiral shapes have also been studied: for example, Sorom randomised 48 patients to either a graft with a flared outflow expansion or a traditional graft, finding improved patency at 1 year with the modified graft (64 vs 32%, p = 0.039) [28]. Grafts made of biological materials, such as bovine carotid artery have also been studied: in an industry funded trial 53 patients were randomised to bovine carotid artery or traditional graft. There was no real difference in secondary patency rates, however bovine grafts did have a lower rate of thrombosis and better 1-year primary patency (61 vs 21%, p = 0.001) [29]. Although promising, study numbers are too small for reliable conclusions and biologic grafts are more costly, though some clinicians feel they have a role when the risk of infection is high.

AV access may also be formed in the thigh - this is often but not always in the context of central venous stenosis. Most frequently grafts are inserted, but fistulas may also be formed, by transposition of either the femoral vein, or less commonly the great saphenous vein. Perhaps the most helpful study is a meta-analysis of 782 access formations (92% grafts) across 15 observational studies published between 1988 and 2006 [30]. By far the commonest procedure was the upper-thigh graft (N = 660) which achieved 1-year primary and secondary patency 48% and 69% respectively, not very different from grafts in the arm. Mid-thigh grafts (N = 60) performed similarly with patencies 43% and 67%. Femoral vein transposition fistulas were both more durable, achieving primary and secondary patency 83% and 93%, and less prone to infection (2% v 18% for grafts) though more likely to lead to steal syndrome (21% v 7% for grafts). Very few publications report outcome with great saphenous vein loops, which are regarded as having poor patency [30] though one single centre study reported 70% patency at 12 months [31].

### Surgical and anaesthetic technique

Surgical practice in fistula formation has evolved conservatively: although variation necessarily exists due to differences in patient anatomy, major divergences in practice are uncommon, and only a few alternatives have been compared in interventional studies. Two types of vessel configuration may be used: the original fistula developed by Brescia and Cimino was formed by side-to-side anastomosis between radial artery and cephalic vein at the wrist. But venous hypertension, which may be associated with hand swelling or discomfort, is less common when using an end-vein to side-artery anastomosis, in which the distal vein is ligated, and this has now become the more common approach at all locations. Both approaches are still used however, and are equally successful according to small studies: Mozaffar randomised 60 patients to fistula formation by side-to-side or end-to-side approach, finding similar rates of primary failure at 6 months (20 vs 17%) [32].

The brachio-basilic fistula uses a deep vein, which requires elevation before it can be needled, and this can either be done at the same time as the anastomosis in a single operation, or at a subsequent ‘second stage’ operation. Practice variation therefore exists though the two-stage approach is perhaps more common, despite being less convenient for patients. In a meta-analysis of 2 randomised and 10 cohort studies, comprising 1136 brachio-basilic fistulas, split evenly between the single and two-stage approach, patency at 2 years was better after two-stage formation (RR 2.50, 95%CI 1.66–3.74) possibly due to reduced thrombosis, though needling was delayed by an average of 30 days [33].

Regardless of operative technique, several studies point towards a relationship between surgical experience and outcome. Variation between individual surgeons has been described, for example in an Austrian study of 108 fistulas, patency at one year ranged from 34% to 69% between the 7 surgeons involved [34]. Between institutions variation has also been described: studying 395 fistula formations in 11 centres, primary failure ranged from 8% to 50%, being significantly worse in 6 centres [35]. However, these studies did not demonstrate a relationship with experience and employed suboptimal statistical methods, for example observing group variation and selecting the extremes for pairwise comparison.

In the Dialysis Outcomes and Practice Patterns Study, Saran reported on questionnaires received from access surgeons at 222 facilities in 12 countries [36]. The range of fistula experience during training was wide, from 16 in the USA to 426 in Germany (132 in the UK) and facility fistula to graft ratio was predicted by both the number of accesses formed during training, and the fistula to graft ratio of the training experience. Separating facilities by tertiles of training fistulas, with cut offs at 25 and 75 fistulas, the lowest tertile was associated with significantly shorter primary and secondary patency at that facility, suggesting a clear relationship between training experience and fistula outcome, with a possible threshold-type effect. These studies support a concept of vascular access surgery which places value on experience, favouring allocation of work to those with subspecialty interest.

Unusually amongst surgical procedures, it appears that the choice of anaesthesia may influence clinical outcome. General anaesthesia (GA) is not suitable for older or comorbid patients, in whom it carries increased risks, and is avoided altogether in many countries. Local anaesthesia (LA) is sufficient and cheap, and the most common type in the UK, but regional anaesthesia (RA), though specialist expertise is needed, may lead to improved fistula outcome. In a large US registry study, Levin reported outcome in a cohort of 3527 brachiocephalic fistula formations, split roughly evenly between GA, LA and RA (30, 38 and 33% respectively). Compared to LA and RA combined, fistula utilisation was lower at 3 months after GA (OR 0.39, 95%CI 0.25–0.61, p < 0.001), though primary patency at 1 year was similar [37].

Support for RA comes in particular from one study in which 126 patients undergoing single-stage fistula formation, were randomly allocated to LA or RA (brachial plexus block) with much better 3-month primary patency observed after RA (84 v 62%, OR 3.3, 95%CI 1.4–7.6, p = 0.005) [38]. Meta-analysis also favours RA, though with a smaller effect size: in 870 fistula formations, from six randomised studies and one cohort, RA was associated with improved haemodynamics and primary patency (RR 1.2, 95%CI 1.1–1.4, p = 0.001) [39]. Evidence remains insufficient however to recommend an intervention with significant cost and expertise implications. Within the UK, the ACCESs study is a large randomised controlled trial currently under recruitment, which plans to investigate one-year functional patency and cost-effectiveness of RA versus LA for fistula formation (ISRCTN No:14153938).

RA is usually achieved via the brachial plexus block: for those unfamiliar with this several high quality reviews are available [40]. Complications of RA can include reflex bradycardia and hemi-diaphragm paresis, but in the modern ultrasound-guided era, serious complications such as pneumothorax and long-term neuropathy are rare (both < 1/1000).

### Maturation

Once formed, regular assessment of AV access is important to detect complications, including dysfunction which may otherwise lead to access failure. Basic physical assessment using a ‘look, feel and listen’ approach is a simple and effective way to monitor the AV access and detect dysfunction: observing the arm, palpating the vessel and listening for the ‘bruit’ with a stethoscope [41, 42]. In the maturing fistula, physical assessment is more challenging, but is still the main method for determining maturation status and initiating cannulation. Physical assessment alone is 81% accurate in predicting maturity [3], and can be supplemented by ultrasound when the vessel cannot be easily palpated [42–44], with vein diameter being the most predictive ultrasound parameter. Whilst prompt detection of problems seems desirable, the effectiveness of angioplasty for maturation failure is not clear [45, 46] and Allon found that closer surgical monitoring after fistula formation led to *delayed* cannulation, which they hypothesise was due to unnecessary diagnostic testing [47].

The optimum timing for maturity assessment is uncertain, and may depend on whether the assessment is positive or negative. If physical assessment is unable to confirm maturation, ultrasound assessment has been suggested at 4 weeks [3] or 6 weeks [48], though a limited number of time-points were actually assessed in these studies. But if the fistula seems mature by physical assessment, then cannulation may be appropriate any time after 2 weeks: although a DOPPS study found increased fistula failure with cannulation before 2 weeks [49], others found no difference in long term fistula outcome between those cannulated before or after 4 weeks from formation [50].

The criteria by which one may determine maturation are unclear. Whilst many quote the ‘Rules of 6’ from previous KDOQI guidelines, these criteria have no clear evidence base, and may be too conservative. In the Haemodialysis Fistula Maturation study Robbin identified fistula flow, diameter and depth as predictors of successful cannulation, but did not recommend specific thresholds, instead suggesting a prediction model based on continuous relationships between ultrasound measurements and maturation: for example each 1 mm increase in fistula diameter increased maturation by 10% (95%CI 10–34%) whereas each 1 mm increase in fistula depth decreased maturation by 24% (95%CI 16–31%) [48]. Smaller studies have indicated that fistula diameters between 4 and 5 mm may be cannulated successfully [44, 51]. Conclusions of a scoping review suggest that diameter greater than 4 mm combined with flow greater than 500 ml/min should be used to indicate fistula maturity [52].

No interventions are known to improve fistula maturation, but three possibilities have been studied to some extent. Surprisingly little literature discusses the effect of blood pressure or hydration on maturation, though both Remuzzi [53] and Siddiqui [54] discuss the importance of maintaining uniform pressure and flow through the fistula to promote maturation, and the hypothesis that low flow might increase the risk of failure seems very plausible. In a retrospective study of 1051 fistula formations, of which 4% had thrombosed by one week, Yan found that early thrombosis was associated with lower pre-operative mean arterial pressure, though the blood pressure difference between groups was small (141/83 v 135/80 mmHg, p = 0.04) [55]. Lower pre-operative blood pressure was also predictive of cannulation failure at 4 months in a prospective observation of 224 radio-cephalic fistula formations [56]. In a secondary analysis of the FAVOURED study (see below) in which thrombosis or cannulation failure occurred by 12 months in 47% of 536 participants undergoing fistula formation, a linear relationship between blood pressure and poor outcome was observed which persisted in adjusted models (OR 1.23 per 10 mmHg decrease in diastolic blood pressure, 95%CI 1.08–1.41) [57]. Although limited, data therefore support the relevance of adequate blood pressure rather than adequate hydration, though either medication or target weight may be appropriate for review. However, any intervention aiming to improve fistula outcome by increasing blood pressure temporarily, would need to be started pre-operatively.

Far infrared therapy involves placing fistulas under an infrared lamp for part of each dialysis session, which increases fistula size and blood flow over time, through mechanisms which are not fully understood [58]. First studied in Taiwan in 182 haemodialysis patients dialysing for at least 6 months via an established fistula, by the end of one year the treatment group exhibited greater fistula blood flow (by 71 ml/min) accompanied by greater unassisted patency (86 v 68%, p < 0.01) [59]. It has also been studied as a method to improve maturation: in 122 pre-dialysis patients undergoing fistula formation, greater 1-year unassisted patency was observed in those randomised to receive far infrared therapy during the year (87 v 70%, p = 0.01) [60]. The intervention is cumulatively costly however, requiring 40 minute treatments thrice weekly over a year, and although promising, these data require further confirmation in the maturation setting.

Routine administration of medications which might improve fistula maturation, have generally been disappointing, with no clear efficacy so far demonstrated. In the multinational FAVOURED study, Irish randomised 567 pre-dialysis patients undergoing fistula formation to fish oil or placebo, and aspirin or placebo, in a 2 × 2 design. Treatments were started the day before surgery and continued for 3 months, but by 12 months, similar rates of thrombosis or cannulation failure were seen between fish oil and placebo (RR 1.03, 95%CI 0.86–1.23) and between aspirin and placebo (RR 1.05, 95%CI 0.84–1.31) [61]. In a large high quality study, Dember randomised 877 patients undergoing fistula formation (46% before dialysis initiation) stratified by location (radio-cephalic, brachio-cephalic or brachio-basilic) to clopidogrel for 6 weeks versus placebo (previously prescribed antiplatelet agents were stopped). Patients were only included if the fistula was clinically patent post-operatively, with treatment started within 24 hours of surgery. Thrombosis before 6 weeks was reduced by one third in the intervention group (RR 0.63, 95%CI 0.46–0.97) but subsequent ‘suitability failure’ (those either abandoned or non-mature) was not changed (62 v 60%, RR 1.05, 95%CI 0.94–1.17) [62]. Though post-operative thrombosis was reduced clinical outcomes were no different, the implication being that the fistulas saved from thrombosis were destined for maturation failure anyway. A meta-analysis of 3 small short studies examining use of ticlodipine indicated improved maturation at one month (OR 0.45, 95%CI 0.25–0.85, p = 0.009) [63], providing insufficient evidence for widespread adoption, though antiplatelet use is favoured by some clinicians. One trial of warfarin for maturation was discontinued early due to bleeding events, and a Cochrane review summarises these studies [63].

### Cannulation

Cannulation should begin with an assessment of the access: a ‘look, feel and listen’ assessment is easy to complete prior to each cannulation to ascertain if the access is healthy or if there is cause for concern. Utility evidence is lacking, but healthcare professionals believe that prior assessment facilitates successful cannulation, as information gained may modify the procedure [64, 65]. This assessment is important to detect problems with the access and facilitate accurate cannulation, whether it is a healthcare professional, carer or patient who cannulates the access. Carers and patients who cannulate should be taught how to assess the access, using the ‘look, listen and feel’ approach. Some patients or carers may struggle with this assessment if they have reduced sensation in their hands, limiting the feel assessment, or they cannot hear through a stethoscope. This should not create a barrier to self-cannulation, but if patients or carers who cannulate struggle with these elements of the assessment, then they may need to be performed by a healthcare professional on a less frequent basis.

As discussed it is worth remembering that fistulas and grafts are formed for the sole purpose of cannulation to enable haemodialysis. In achieving this, cannulation itself has two key goals, which may sometimes compete: the first is cannulation success at dialysis (achieving each day’s dialysis with minimal symptoms, first-time success and no complication, ie. avoiding miscannulation and infection) and the second is maintaining long term fistula health (preventing the development of stenosis that can lead to access thrombosis, aneurysm or ulceration due to repeated vessel trauma) [66, 67]. Both these issues are important to the experience of patients, who view cannulation as an unpleasant procedure balanced with the sole but significant benefit of achieving haemodialysis. Negative patient experiences include needling pain, fear of miscannulation, dependency, vulnerability and anxiety [7–11], contributing to the avoidance of AV access in some patients [4, 5, 11]. Within the UK, cannulation is a key target for improvement, with annual Patient Reported Outcome Measures regularly citing cannulation as a key area of concern for patients [68–71].

Preparation of patients for cannulation is helpful in reducing anxiety and improving the experience of needling. In paediatric settings this is routinely available but the need for it is often unanticipated in adults. The British Renal Society (BRS) and Vascular Access Society of Britain and Ireland (VASBI) needling recommendations [72] provide advice and further detail on how best to prepare patients for cannulation of their access, using the expertise developed in paediatric settings. This includes providing information prior to the first cannulation, techniques to de-sensitise patients to needles, providing a calm environment, having a cannulation care plan and use of distraction techniques during needling. Interventions to reduce anxiety and pain during needling may also include local anaesthesia during needling, music therapy and other relaxation techniques, though as of yet there are no studies that demonstrate efficacy of these interventions [9].

As the fistula was developed through the 1970’s replacing shunts for dialysis access, a standard cannulation practice was established by which needle sites were varied to allow the punctured skin and vein wall to heal well before repuncture. The problems of aneurysmal deformation and needle site ulceration became well known early on [73], which led to three original cannulation techniques being described: ‘rope ladder’ involving systematic progression up and down the vessel with an aim of reducing the frequency of cannulation per cm squared; ‘area puncture’ where cannulation sites cover small areas; and ‘buttonhole’ where the needle is inserted in exactly the same site each time [74]. Kronung recommended the avoidance of area puncture, as it was associated with stenosis development, thus promoting the use of rope ladder or buttonhole. Since it’s inception, buttonhole has always been avoided in graft cannulation, due to the risk of infection and graft degradation. Grafts are straight and usually of sufficient length to allow easy rope ladder needling, so as no further evidence is available, only rope ladder is recommended for graft cannulation.

Recently effort has been focussed on whether buttonhole or rope ladder is the optimum technique for cannulation of fistulas, with divergent reviews favouring buttonhole [75], or restricting buttonhole to difficult fistulas [76, 77]. Randomised controlled trials that compare buttonhole and rope ladder demonstrate varying results with flaws in the study design [78]. These studies have been focussed on in-centre cannulation performed by healthcare professionals. As there is a belief that buttonhole is beneficial for patients who cannulate themselves, making the cannulation procedure easier and safer, Huang performed a pilot randomised controlled trial to compare the two techniques in the home haemodialysis population. They were unable to complete the study due to patient preference for buttonhole [79], though it was unclear whether this was driven by patient or healthcare provider preference. Therefore, there is no current consensus or definitive study to determine whether buttonhole or rope ladder is optimal: with no universally optimum technique, the selection between rope ladder and buttonhole cannulation should be individualised. The BRS and VASBI needling recommendations [72] provide further detail on advice on how to do this, but in particular, provider preference should not be the sole driver of needling practice. Neither, of course, should provider inexperience be limiting, and units should therefore allow sufficient training resource to establish and maintain expertise in all cannulation types.

Discussions on rope ladder or buttonhole cannulation often neglect area puncture. For a long time, area puncture has been associated with aneurysm and stenosis development, and is widely believed to shorten the lifespan of the access. Clinicians commonly see aneurysm development at sites of area puncture, though research evidence is limited, and it should be acknowledged that reverse causation may play a role, since it is harder to achieve rope ladder needling in fistulas which are aneurysmal. But prospective studies also lend support to the view that area puncture causes access failure: in a European study of cannulation practices, 7058 patients were followed for up to 3 years, during which 1485 required new access formation (21%). Compared to rope ladder, area needling was associated with earlier access failure (HR 1.12, 95%CI 1.00–1.27) [80]. There is general agreement therefore that area puncture should be avoided where possible, to prevent access complications and failure.

However, despite the shared concern of healthcare professionals and the aim in dialysis units to minimise it, area puncture continues to be the most prevalent cannulation technique: in Parisotto’s multicentre study of 10 807 cannulation episodes, area puncture was observed in 66%, with rope ladder (28%) and buttonhole (6%) forming a smaller group [80]. Some area puncture should be expected: rope ladder needling requires an adequate length of fistula accessible to cannulation, so it may not be achievable in short fistulas and those which have developed aneurysmal or other degeneration. But rope ladder may also be a more difficult technique, being associated with more miscannulation than area needling (OR 1.63, 95%CI 1.28–2.07) [81], and short term incentives may therefore encourage patients or nurses to favour established sites, and patients to favour cannulators who prioritise today’s success over future access. We favour routine promotion of rope ladder or buttonhole needling, but acknowledge uncertainties in the evidence base, and the existence of patients for whom area puncture is the best or only option. Since the chief downside to area puncture is fistula failure, it is clearly preferable to fistula abandonment, and may effectively extend the fistula’s functional duration. The BRS and VASBI needling recommendations include details (outside the scope of this guideline) on how to avoid area puncture where possible, and where it is not possible, how to use it safely [72].

Whilst many units claim to avoid area puncture, cannulation techniques are loosely defined with blurred lines between rope ladder and area puncture. The original rope ladder description requires vessel cannulation along a significant length of the vessel to allow adequate rotation of sites. However, many interpret variation of needle sites over short segments as ‘rope ladder’ rather than area puncture. Potentially much of the disparity in results in cannulation studies could be related to this lack of definition, making it unclear whether buttonhole is compared to rope ladder or area puncture [78]. To correct this, the BRS and VASBI needling recommendations (2018) provide detailed definitions of each technique, which have been adopted for this guideline:

- Rope ladder is defined by a systematic progression of needle sites along the fistula or graft, progressing by 5–10 mm each session, restarting at the beginning once the end is reached. To be classified as rope ladder and not area puncture, needling sites should cover at least 8 cm (combined) or 5 cm (for each needle) if the arterial and venous needle are on separate segments of the vessel.
- Area puncture defines sites which are varied but within smaller regions, without a systematic linear plan.
- Buttonhole refers to needling in exactly the same place each session. At the start of each cannulation, the scab from the previous cannulation is removed. Sharp needles are used initially over several weeks to develop a track, which can then be accessed with blunt needles.

Another priority of cannulation, beyond preserving the function of the fistula or graft, is to avoid miscannulation. Miscannulation refers to an unsuccessful cannulation attempt, where there is more than one attempt to insert either the arterial or venous needle (or both). Miscannulation is one of the most frequent cannulation complications, occurring in 4% of dialysis sessions, and more common in new fistulas [82, 83] though the rate alone may underestimate patient impact, since one miscannulation event may entail up to five further attempts before cannulation is achieved. Two-thirds of patients experience miscannulation when establishing a new fistula [84], and some patients experience miscannulation frequently: over 6 months, Van Loon found that 37% of patients with a new fistula and 19% of patients with a new graft had more than ten missed cannulations [85].

Miscannulation often leads to pain and bruising, though the lower rate of reported haematomas (5% per patient-year) suggests that not all miscannulation leads to complications [86]. Haematomas lead to diagnostic and surgical procedures, and miscannulation may also lead to abandoned dialysis sessions and access failure. Haematomas are also associated with maturation failure [47], though this observation may reflect the reverse effect of non-maturation on needling difficulty. Miscannulation is of concern to patients, contributing to a ‘bad’ haemodialysis treatment, with increased pain, delayed dialysis initiation, and sometimes persistent haematoma [10]. Wilson and Harwood found unsurprisingly that for patients ‘successful cannulation’ requires first-time success with both needles, but also successful use of the needles for dialysis [9]. The burden that miscannulation causes to patients is therefore easily identifiable.

In order to reduce miscannulation, particularly with new fistulas, two specific strategies have been suggested: ultrasound assistance and single needling. Ultrasound assistance prevents complications of venous catheter insertion [87] and assisted fistula cannulation is promoted by several authors but no study provides a clear evaluation of utility [43]. Two studies used ultrasound to assess the position of needles inserted in the usual (‘blind’) manner: Nalesso (N = 45) and Marticorena (N = 86) both found that many needles were in suboptimal positions, therefore recommending ultrasound guided cannulation [51, 88]. Observational studies cannot be relied on since they often show reverse causality - ultrasound is mostly used for difficult fistulas, so its use is associated with more, rather than less, miscannulation [82].

Another strategy is single needle dialysis, which halves the number of cannulations required, at the cost of reduced dialysis dose (or increased time to achieve the same dose) [84]. A small study (N = 22) found that single needle haemodialysis leads to less miscannulation (1.2 v 2.5 cannulation attempts per dialysis) whilst maintaining acceptable clearance [89]. Regardless of technique, it is likely that the training of cannulators (nursing staff or patients) is also relevant to success. Direct evidence is not available and should not be expected, since studying an untrained cannulator group would be unethical, but circumstantial considerations support the concept and many authors believe that cannulation could be improved. Labriola reported an increased infection risk with buttonhole needling which was overcome by a strict training programme for cannulators, and Chow felt that buttonhole complications were associated with breaches in technique, rather than the technique itself [90, 91]. Despite standardisation and competency frameworks, cannulation practice continues to be driven by provider preference.

One promising initiative is MAGIC (Managing Access by Generating Improvements in Cannulation), a quality improvement supported by KQuIP (Kidney Quality Improvement Partnership), which uses structured education and feedback to improve cannulation [92]. Initial results from the first two regions demonstrated a large reduction in area needling, and it is currently undergoing wider adoption and evaluation. Whilst it may be an assumption that cannulation can be improved by education, it seems obvious that such a difficult and important procedure should only be performed those who are competent.

## Guideline 4. AV access problems

4.1  We suggest a shared decision in the management of AV access complications, taking into account clinical severity, treatability, alternative access options and patient priorities [2C]
4.2  We recommend intervention for patients with radiologically significant stenosis and clinical features of AV access dysfunction [1B]
4.3  We suggest endovascular treatment as the initial approach for non-complex AV access stenosis, using high-pressure balloons (up to 40 atm) where necessary to overcome AV access stenosis [2C]
4.4  We recommend covered stents for the treatment of stenosis at the graft-vein outflow anastomosis, following adequate balloon dilation [1C]
4.5  We recommend either an endovascular or surgical approach to salvage of thrombosed access based on local expertise. Surgical approaches should be followed by treatment of the underlying culprit stenosis [1C]
4.6  We recommend regular assessment of AV access aneurysms, with intervention dependent on symptoms, access function and the risk of spontaneous bleeding [1C]
4.7  We suggest surgical repair as the main approach to aneurysm treatment, combined with inflow reduction or endovascular treatment of downstream stenosis where appropriate [2D]
4.8  We suggest that an awareness of steal syndrome, including risk factors, clinical consequences and indications for urgent treatment, is important for all clinicians caring for haemodialysis patients [2C]
4.9  We suggest that mild steal syndrome should be managed conservatively [2C]

## Rationale

A proportion of fistulas and grafts develop dysfunction over time, which may manifest clinically as flow dysfunction, thrombosis, aneurysm, steal syndrome or a high flow state. The incidence of complications varies widely between studies, but in a meta-analysis of 43 cohort studies published between 2001 and 2014, covering 11 374 fistulas, with median follow-up 17 months, thrombosis, steal and aneurysm developed with a yearly incidence of 8.8%, 1.8% and 1.5% respectively [1]. These complications may have multifactorial aetiology, but can all result in loss of dialysis access as well as symptoms and potentially even death [1–4], hence careful and timely management is essential.

A lower incidence of complications has consistently been reported with fistulas, compared to grafts. For example, in a two-centre study, Lok studied access durability and complications in 128 patients with a graft, and 1012 patients with a fistula [5]. Although initial function was achieved more commonly with grafts (81% vs 60%, p < 0.001), subsequent secondary patency was shorter at 24 months, versus 62 months for fistulas (HR 0.56, 95%CI 0.43–0.74), with a greater need for interventions including angioplasty (1.2 vs 0.5 per year, p < 0.001) and thrombectomy (0.36 vs 0.02 per year, p < 0.001). Similarly, in a meta-analysis of 11 studies, Ravani found increased rates of access failure with grafts, reporting relative risks ranging from 1.48 (0.95–2.29) to 4.10 (2.22–7.56) [4].

Clinical evaluation forms the mainstay of the assessment of vascular access dysfunction, with several authors highlighting its value. Asif studied 142 patients referred for angiography, comparing radiological diagnosis with examination findings, such as pulse augmentation (failure of transient fistula compression to augment the pulse indicating inflow stenosis) and arm elevation (failure of arm elevation to reduce fistula fullness indicating outflow stenosis). Examination findings were 85% sensitive and 71% specific for detecting an inflow lesion, and 92% sensitive and 86% specific for detecting an outflow stenosis [6]. Similar support for clinical examination came from Coentrao [7] who also noted the value of specific training in improving the accuracy of clinical skills.

Though some high quality studies are available, the literature on access complications is limited by small study populations, heterogeneity and short term outcomes, leaving many knowledge gaps, so that an evidence-based consensus is not possible for all aspects of management. One consistent theme is uncertainty of outcome, suggesting the need for pragmatic shared decisions taking into account clinical risk (e.g. of access loss or haemorrhage), likely treatment outcome and patient preference. Although in many cases the access may be successfully salvaged, it is often helpful if a back-up plan for alternative access is also discussed within the multidisciplinary team, and this is consistent with the KDIGO concept of a ‘life plan’ for vascular access.

### Stenosis

Significant AV access circuit stenosis can manifest broadly as disorders of inflow (presenting with needling difficulty, inability to achieve flow or inadequate dialysis) or of outflow (presenting with arm swelling, prolonged needle site bleeding or inadequate dialysis). Flow dysfunction may therefore be problematic immediately, leading to symptoms and treatment burden, but importantly also it leads to a cumulative risk of access thrombosis, a serious event which leads to further treatment burden (unplanned admission, temporary access), clinical risk (delayed dialysis) and sometimes loss of access: thrombectomy is not always attempted, not always successful, and recurrent thrombosis is common.

The pathophysiology of AV access circuit stenosis is incompletely understood, involving fibromuscular proliferation and neointimal hyperplasia, thought to be driven by flow turbulence and wall shear stress [8]. Angiography is usually the initial step since it combines accurate anatomic diagnosis with concurrent treatment, being therefore logical and convenient, and with advances in endovascular techniques these are now the mainstay of management.

### Selection for angiography

Angiography is usually triggered when access stenosis is suspected due to clinical features (dialysis problems or examination findings) which may also be supported by haemodynamic monitoring (venous needle pressures or access flow). The role of routine surveillance of AV access, with angiography triggered by haemodynamic monitoring or ultrasound (without clinical evidence of dysfunction) is controversial, but has been recommended in previous guidelines, such as NKF-DOQI in 1997 [9]. In an early study, Besarab described a 6-year quality improvement program in a single centre of 180 patients (with 30% yearly patient turnover), which saw increasing use of venous pressure to prompt angiography, and reducing radiological thresholds for stenosis treatment [10]. The use of angioplasty increased from less than 0.1 to 0.25 per patient-year, associated with a reduction in thrombosis and 79% reduction in access failure.

The benefit was inconsistent in randomised studies, however, summarised by Tonelli’s meta-analysis of angiography triggered by reduced blood flow or ultrasound screening. In patients with fistulas (4 studies, 360 patients) reduced thrombosis was seen (RR 0.47, 95%CI 0.28–0.77) but without a clear reduction in access failure (RR 0.65, 95%CI 0.28–1.51) [11]. In patients with grafts (7 studies, 446 patients) there was no clear reduction in either thrombosis (RR 0.94, 95%CI 0.77–1.16) or access failure (RR 1.08, 95%CI 0.83–1.40). In a larger subsequent meta-analysis in which fistulas and grafts were pooled, Ravani found a modest reduction in thrombosis (RR 0.79, 95%CI 0.65–0.97) but less clear prevention from access loss (RR 0.81, 95%CI 0.65–1.02) [12]. Paulson provides a helpful analysis in terms of WHO principles for surveillance programs [13], describing the concept as a ‘false paradigm’, and access surveillance has disappeared from more recent guidelines.

### Angioplasty and outcome

There is no clear definition of the anatomic criteria for stenosis, but a clinically relevant stenosis reduces the luminal diameter by at least half, and usually much more than this, since lesser degrees of stenosis are not sufficient to be clinically noticeable. In identifying culprit lesions it is generally accepted that > 70% diameter reduction when compared to the adjacent vessel segment represents a significant lesion, though 50% has sometimes been advocated [14] and lower thresholds may be appropriate depending on the severity of clinical dysfunction. Significance of a lesion may also depend on other anatomic factors, such as angulation and absolute (rather than relative) diameter, and the judgement of an experienced radiologist is therefore essential.

Once selected, a stenosis is treated with balloon dilation (fistuloplasty), aiming to disrupt inelastic tissues, and reduce or eliminate the stenosis. Technically successful fistuloplasty is considered to require no more than 30% residual stenosis, but effective treatment often necessitates the use of ‘high-pressure’ balloons (up to 40 atm). Many patients report severe pain associated with angioplasty, and the requirement for analgesia should therefore be anticipated. Regional (e.g. brachial plexus block) or general anaesthesia may allow for better tolerance, depending on anatomical location [15], but provision may be dependent on locally available expertise.

Technical success does not always imply clinical success, and the latter has both short term and long term aspects. Whilst angioplasty frequently resolves current dialysis problems, the durability of such effects is more variable, with recurrent stenosis and later access loss sometimes seen. Typical outcome is dependent on the type of lesion: as examples, primary patency of graft-vein outflow stenosis 6 months after balloon angioplasty has been reported at 51% [16], and primary patency of cephalic arch stenosis 6 months after balloon angioplasty in brachiocephalic fistulas has been reported at 81% [17].

These estimates come from small studies however, without a control group (which by modern practice would seem an unethical study group): they may not reflect outcomes in ordinary clinical practice therefore, where the benefit of fistuloplasty is harder to quantify. Helpful insight is provided by a large US database linkage study, in which Chan used a case-control design to estimate the benefit of fistuloplasty in preventing access loss, defining ‘cases’ as patients undergoing their first AV access intervention (N = 4181), selecting 8 non-intervention controls for each case, matched for access type (fistula or graft), access age, access flow (mean and slope over the previous 2 months) and dialysis adequacy [18]. By one year after intervention, half of all accesses had failed, with no apparent intervention advantage (in fact a slightly higher access failure rate at 55% vs 48% in non-intervention controls). The selection criteria were unknown however, with the intervention group containing surveillance angiograms, and two subgroups emerged in whom a clear benefit for intervention was seen: the lowest quartiles of both access age (median(IQR) 0.4(0.2–1.0) years) and access flow (median(IQR) 672(439–1035)ml/min). No differences were seen with respect to patient characteristics or access type, and serious complications (contusion, vessel injury and embolism) were seen in only 1%. This study therefore confirms the benefit of fistuloplasty but highlights also its situation-specific nature, reiterating the importance of clinical criteria in selecting patients.

### The role of stents and drug-coated balloons

Though balloon dilation alone is usually successful, subsequent stent insertion is helpful in cases where rapid elastic recoil occurs, and stents may be better at preventing recurrent stenosis, at least in specific anatomic circumstances. Care must be taken to avoid loss of needling area or occlusion of branch vessels, and covered rather than bare metal stents (termed ‘stent grafts’ in some literature) are usually used. Specific locations are more prone to recurrent stenosis after angioplasty, the two commonest being the graft-vein outflow anastomosis of grafts and the cephalic arch of brachiocephalic fistulas, with evidence best supporting the use of covered stents for graft outflow stenosis.

Haskal studied 190 patients with graft outflow stenosis, randomly assigned to covered stent placement versus balloon angioplasty alone, with follow-up including angiography as indicated clinically and at 2 and 6 months [19]. Primary patency (freedom from > 50% stenosis) of the treatment area at 6 months was greater in the stent group (51% vs 23%, p < 0.001), with no difference in procedural adverse events, which were uncommon. Other studies corroborate this finding: Vesely studied 293 patients with stenosis of graft outflow, randomly assigned to covered stent versus balloon angioplasty alone, observing improved target lesion primary patency with covered stents at 6 months (52 v 34%, p = 0.006) [20]. And in a meta-analysis of 3 randomised and 5 cohort studies, Kouvelos reported outcomes in 1051 patients with graft dysfunction, with 98% of lesions at the graft-vein outflow [21]. Patients were evenly split between balloon-only and balloon-then-stent groups, with covered rather than bare metal stents used most frequently (88%), and at 6 months, loss of patency was seen less often after stent placement (47% vs 67%, OR 0.42, 95%CI 0.31–0.57).

Improved patency of cephalic arch stenosis has also been reported after treatment with a covered stent, though some of the studies are quite small: for example Rajan studied 14 patients with cephalic arch stenosis randomly assigned to covered stent placement (N = 9) versus balloon angioplasty alone (N = 5) finding all of the covered stents but none of the balloon-only treatments patent at 6 months [22]. The largest study is a meta-analysis in which D’cruz included 457 patients undergoing treatment for cephalic arch stenosis in 11 studies, of which 3 were randomised (34 patients) and 8 observational (423 patients) [23]. At 6 months primary patency with covered stents, bare metal stents and balloon-only treatment was 83, 52 and 23% respectively, with least patency loss observed with covered stents (RR 0.30 v bare metal stents, 95%CI 0.19–0.41, RR 0.59 v balloon-only, 95%CI 0.50–0.66). Secondary patency at 12 months similarly differed between treatment types at 98, 85 and 68% respectively. Stents may reasonably be employed in other types of AV access stenosis, for example for early stenosis recurrence, but when compared, bare metal stents have consistently been outperformed by covered stents, with the former therefore largely being abandoned.

The effect of balloon angioplasty may also be more durable if drug-coated balloons are used to deliver an anti-proliferative agent directly to the fistula wall. Paclitaxel (a cancer drug which targets the cytoskeleton and blocks cell division) is the most studied agent, but reports in the literature vary with some trials demonstrating an advantage and others showing no beneficial effect. Trerotola randomised 285 patients with a dysfunctional fistula to either a paclitaxel-coated (2 μg/mm^2^) or an uncoated balloon (deployed after successful stenosis treatment with a plain balloon) [24]. In the study’s main outcome, 6-month primary patency, there was no clear difference (71% vs 63%, p = 0.06) though in a follow-on study possible effects on longer term outcomes were reported including 1-year (44% vs 36%, p = 0.04) and 2-year primary patency (27% vs 24%, p = 0.09).

Clearer support for paclitaxel balloons came from Lookstein, who randomised 330 patients with > 50% fistula stenosis to either a paclitaxel-coated (3.5 μg/mm^2^) or an uncoated balloon, reporting improved patency at 6 months (82% vs 59%, p < 0.001) [25]. Benefits extended also to 1 year patency (64% vs 44%, p < 0.001) along with a reduced need for re-intervention (0.35 vs 0.54 py, p = 0.001). However, no benefit was seen in a large UK investigator-led study: Karunanithy randomised 212 patients with a dysfunctional fistula and a single (>50%) stenosis to either a paclitaxel-coated (2 μg/mm^2^) or an uncoated balloon, with no evidence of benefit (HR 1.18 for time to loss of target lesion primary patency, 95%CI 0.78–1.79) [26]. Taken together therefore, these studies provide insufficient rationale for the routine use of drug-coated balloons for every stenosis associated with AV access. However, since drug-coated balloons have no real patient disadvantage (though treatment time and cost are increased) their selective use for recurrent lesions is considered appropriate by some clinicians [25–28].

### Thrombosis

The most important consequence of AV access stenosis is thrombotic occlusion, and in most cases of access circuit occlusion there is a haemodynamically significant culprit stenosis. Fistula salvage therefore needs to address both the thrombus and any stenosis which may have been contributory, and a review of prior interventions, recent access flow rates, and needle pressures, is helpful in making management decisions.

Historically open surgical thrombectomy (with or without treatment of the underlying stenosis) was the mainstay of treatment, and continues to be the dominant approach in many centres nationally, achieving initial access salvage in just under two-thirds of cases, depending on access type. Ghaffarian studied the effectiveness of this approach in 209 cases of access thrombosis (35% in fistulas and 65% in grafts) [29]. Fistula thrombectomy was followed by angioplasty in 57% of cases and surgical revision in 9%, achieving successful salvage in 56% of forearm fistulas and 70% of upper arm fistulas. Graft thrombectomy was more often followed by further intervention (angioplasty in 74% and surgical revision in 18%) but achieved successful salvage with similar frequency (63%). Recurrent events were frequent however, impacting on longer term outcomes: by one year, 43% of forearm fistulas, 44% of upper arm fistulas and 31% of grafts remained patent, with half of fistulas and most grafts requiring further procedures to achieve this.

More recently, advanced endovascular techniques have allowed safe extraction of thrombus with simultaneous treatment of associated stenosis, in a single procedure. Some studies have reported high success rates with this approach: for example, Tan studied 294 cases of access thrombosis (53% in fistulas and 47% in grafts) reporting initially successful salvage in 91% of fistulas and 96% of grafts [30]. Recurrences remain frequent following this approach however: 67% of fistulas and 60% of grafts remained patent at 6 months, with authors noting poorer patency in those with a recent (within 3 months) prior event.

Increasingly, centres have adopted an endovascular approach, or on occasion a hybrid approach, for thrombosed access salvage [31], but with heterogeneity in (particularly endovascular) techniques and few comparative studies, the optimum approach is not clearly established. In a meta-analysis of 8 randomised and 2 cohort studies, Chan reported outcome after 1072 graft thrombectomy episodes, 63% treated initially surgically (thrombectomy, followed by anastomosis revision including interposition graft or endovascular angioplasty) and 37% treated with an endovascular approach alone (thrombolysis and angioplasty) [31]. Technical failure appeared more common in the endovascular group (27% vs 13%, p = 0.03) though outcomes after one month were similar, with primary (without recurrent event) patency 61% and 66%, and secondary patency 74% and 73% in the endovascular and surgical groups respectively. Similarly at 3 months there was no difference between the groups, but by one year primary patency was lower in the endovascular group (RR 0.82, 95%CI 0.75–0.88). Contributory studies were variable in their definitions however, with surgical success in the largest contributory study [32] defined to include formation of new access, only grafts included, and most importantly, few details provided on the factors influencing treatment selection.

On available data therefore, it seems that in those cases where either is thought appropriate, endovascular-only and surgical-first approaches are broadly equivalent in outcome, though this is also dependent on centre experience. As a single procedure, the endovascular-only approach is more convenient for patients. One element of treatment seems reasonably clear: in a before-after study of 329 cases in which early endovascular thrombectomy was facilitated (with the proportion achieved within 24 hours improving from 55% to 93%), Hsieh reported a clear improvement in 3-month patency in fistulas (68% vs 50%, p = 0.03) but perhaps not grafts (50% vs 46%, p = 0.65) [33]. Timely treatment is therefore not only better for patients (less delayed dialysis, less temporary access, more convenient) but for fistulas in particular, it is also more likely to work.

### Aneurysm

AV access aneurysms may occur adjacent to the arterial anastomosis, or more commonly along the cannulation segment, and may be focal (with one or two rounded expansions in an otherwise normal fistula) or diffuse (a sausage-like enlargement of most of the fistula). A diameter over 18 mm is commonly used in literature to define AV access aneurysm, though in clinical practice this cutoff is less important than the associated features [34]. A true aneurysm is a dilated region contained within the fistula wall, whereas a pseudoaneurysm (more common with grafts) is a leak through the wall, contained by connective tissue outside the access.

Cannulation trauma, particularly when repeated in a densely cannulated area, is believed to be the dominant causative factor, and is the main rationale for favouring rope-ladder or buttonhole over area cannulation. However, the occasional development of aneurysms in fistulas which have never been cannulated emphasises the role of fistula pressure, from either high flow or downstream stenosis, in causation: Rajput described 89 patients requiring intervention for dysfunctional aneurysmal fistulas, of which 69 (78%) were found to have a downstream stenosis [35]. Those associated with stenosis were more recently formed than those without (4.1 vs 6.4 years) suggesting a causative role in aneurysm development. Coexistance of causes is common, and area cannulation and downstream stenosis may both contribute to the development of aneurysms. In AV grafts, repeated cannulation and loss of graft integrity over time are the most likely causes of pseudoaneurysms.

Aneurysm development often leads to cannulation difficulty since adjacent fistula segments may be distorted and inaccessible, limiting the length available for optimal cannulation technique. In addition, aneurysmal change is often associated with atrophy in areas of overlying skin which may become thin, shiny, depigmented and hairless: such areas heal poorly and should not be cannulated. Optimal cannulation technique (as discussed in Chapter 3) is therefore important from the outset, since rope-ladder cannulation becomes more difficult once aneurysmal change has started.

But in addition to problems during dialysis, aneurysms may be uncomfortable or unsightly, and most importantly, lead to an increased risk of rupture and life-threatening haemorrhage. Although rare, the actual incidence of access haemorrhage is unknown due to inconsistent reporting. In a study of 1581 fatalities in dialysis patients coded as ‘haemorrhage of vascular access’ and 71 coded as ‘haemorrhage of dialysis circuit’, Ellingson estimated that 0.4% of all US haemodialysis deaths between 2000 and 2006 were caused by access or dialysis circuit haemorrhage [36]. In subgroup analysis, 6% occurred during a dialysis session, 12% were procedural and 79% occurred outside healthcare settings: risk was lower with catheter access (the majority of which were either sessional or procedural) and greater with graft access and those with a recent access complication. A similar incidence was reported in an Australian study, which estimated a 6-fold increased risk with grafts, and highlighted also the frequency of recent access procedures or skin problems [37].

Like all AV access, aneurysmal AV access should be regularly assessed, but with particular attention to those features which are associated with bleeding risk including erosion (ulcer or scab), rapid growth, prolonged post-dialysis bleeding, and any reports of spontaneous bleeds outside the dialysis unit. Atrophic skin should not be cannulated, either by cannulating the sides of aneurysmal segments where skin is unaffected, or preferably by avoiding aneurysmal segments altogether. Where appropriate, treatment may be directed at causative lesions including downstream stenosis or wide inflow, but where high risk features are present, a surgical approach seems more appropriate, either repairing the fistula by resection of part of the aneurysm wall, or replacing part of the fistula with ‘interposition’ graft.

Literature regarding aneurysm management is largely limited to case series, and it is therefore not possible to make clear recommendations. The most helpful study is a systematic review of 13 published case series, in total describing aneurysm repair in 597 patients, involving fistulas formed between 12 and 144 months previously, 59% of which were in the upper arm [38]. The indication for treatment was most commonly bleeding risk (86%), with high-flow concerns (9%) and patient discomfort (4%) contributing less often. All fistulas were repaired surgically by resecting part of the aneurysm wall, with additional inflow reduction in 7% and endovascular treatment of downstream stenosis in 21%, and cannulation was resumed within 48 hours in 7 studies, and delayed for up to 6 weeks in 6 studies, bridged by catheter access. A pooled complication rate of 11% was estimated, including thrombosis (1.5%), haematoma (2%) and infection (4% of those repaired with prosthetic mesh, N = 95), but repairs were generally durable, with 12-month primary patency 82% (95%CI 69–90%).

Rather than surgically, pseudoaneurysms complicating AV grafts are usually treated by endovascular placement of a covered stent over the pseudoaneurysm origin. Kinning reported 24 covered stents placed for pseudoaneurysm (20 grafts and 4 fistulas): there were 3 early infections leading to graft excision, but 12-month secondary patency was reasonable at 71% (95%CI 81–91%) [39]. Needling through covered stents is not recommended by manufacturers however, so the area available for cannulation is subsequently reduced. In an emergency a covered stent may sometimes be placed as a bridge to surgery.

### Steal syndrome

‘Steal’ is the clinical manifestation of distal ischaemia, developing as a consequence of the diversion of blood into the access, and therefore away from the hand and forearm, after access formation. It usually occurs in the early weeks following AV access formation, but may develop later following balloon angioplasty or as blood flow increases over time.

Steal is often classified according to severity into three grades (mild, moderate or severe) which helpfully align with implications for treatment (Table 1) [40], ranging from no treatment to urgent intervention. Other authors prefer four grades, further separating the severe category according to whether there is tissue loss (ulceration or necrosis) [41]. And several acronyms are used in literature to describe steal syndrome, including Dialysis Access-associated Steal Syndrome (DASS), Haemodialysis Access-Induced Distal Ischaemia (HAIDI) and ArterioVenous Access Ischaemic Steal (AVAIS) [42].

**Table 1:** Clinical grading of steal syndrome

Severity grade	Clinical features	Treatment implication
1 (Mild)	Pale or cool extremity but no pain	No treatment necessary
2 (Moderate)	Pain on exercise or during dialysis	Treatment often needed, may be delayed
3 (Severe)	Pain at rest, distal ulcer or necrosis	Prompt treatment needed

Most steal is seen with brachial artery inflow, with progressively increasing frequency in brachio-cephalic fistulas, grafts and brachio-basilic fistulas, but occasionally steal occurs with forearm access: this is usually associated with flow reversal in the palmar arch, and may be treated with distal radial artery ligation. Anatomic features however are less predictive than patient factors: in a cohort study of 602 participants undergoing fistula formation (76% in the upper arm), after a median(IQR) interval of 2(1–5) months symptomatic steal syndrome developed in 45 (7%), in particular in females (OR 3.17, 95%CI 1.27–7.91), diabetics (OR 13.6, 95%CI 1.81- > 100) and those with coronary disease (OR 2.60, 95%CI 1.03–6.58). Specialised vascular assessment (occlusion plethysmography) was able to determine vessel characteristics (vein capacitance slope) associated with the subsequent development of steal, but routinely available anatomic factors (such as pre-operative vessel diameters, anastomosis size or early post-operative fistula flow) were poorly predictive [43].

The diagnosis is made clinically, according to characteristic features, which may be altered by transient access compression. Doppler ultrasound may demonstrate diastolic flow reversal in the distal artery, but this feature is non-specific, and the role of ultrasound is principally to identify reversible contributory features such as arterial stenosis or high flow access. Non-vascular diagnoses to consider include carpal tunnel syndrome, parathyroid bone disease and arthritis.

There are no trials or comparative studies on which to base treatment recommendations. The need for treatment is dependent on clinical severity (Table 1) with access ligation usually favoured for the most severe cases, since this most quickly and reliably restores perfusion [42]. In most cases the options for treatment depend on associated features: when arterial stenosis is present endovascular balloon dilation may be sufficient, leaving the access alone. Similarly, inflow reduction (eg by surgical post-anastomotic banding) is logical and usually favoured for steal associated with high flow fistulas. For other cases various surgical approaches (known by their acronyms) have been described aiming to resolve the features of steal whilst preserving the access, including Distal Revascularisation and Interval Ligation (DRIL), Proximalisation of Arterial Inflow (PAI) and Revision Using Distal Inflow (RUDI). In Huber’s study in which 45 (7%) of patients developed symptomatic steal, 26 (4%) underwent intervention, including ligation [7], inflow banding [4] and DRIL [13].

DRIL, first described in three patients [44], involves two-stage surgery: firstly using a vein or graft conduit to provide distal perfusion bypassing the anastomosis, and secondly ligating the native artery just distal to the access anastomosis, so that distal perfusion is entirely dependent on the conduit. In a systematic review of 22 case series, Kordzadeh studied 459 DRIL procedures, used to treat steal syndrome occurring 6(1–20) months after access formation including upper arm fistulas (74%), grafts (21%) and forearm fistulas (2%) [45]. The saphenous vein was most commonly used as the conduit (77%), with arm veins (12%) and grafts (11%) used less often, and over a median follow-up of 18 months, primary (without intervention) patency of both bypass and access was achieved in 81%. Bypass thrombosis was most common with grafts, occurring in 43%, and concern over this complication has limited enthusiasm for this procedure, in which the access is perfused by native artery, whereas the hand is perfused by a bypass.

In contrast, RUDI, first described in four patients [46], preserves native artery perfusion of the hand, using a bypss to perfuse the access, anastomosed to a more distal part of the native artery. In a systematic review of 11 studies covering 130 RUDI procedures for steal syndrome (99% in upper arm fistulas), the conduits used to perfuse the access were saphenous vein (63%), arm vein (28%) and graft (9%) [47]. Over a median follow-up of 12 months, primary patency was 82%, with spontaneous access thrombosis in 8%, but ligation was required in the remaining 11% for ongoing steal syndrome, with finger amputation required in two patients. Whilst promising therefore these novel surgical techniques are not without drawbacks, and should be employed with caution and careful patient discussion. These issues highlight the importance of vascular mapping and other aspects of pre-formation assessment, considering patient as well as anatomic factors, with patients at the centre of the decision making process.

## Guideline 5. Dialysis catheter insertion and care

5.1  We recommend routinely favouring the right internal jugular vein for tunnelled haemodialysis catheter insertion, though vessel imaging, AV access location and patient preference may modify site selection [1C]
5.2  We recommend routinely avoiding the subclavian route where alternative veins are available, particularly in children and young adults [1C]
5.3  We recommend real time ultrasound to optimise tunnelled haemodialysis catheter insertion, as well as fluoroscopy for left-sided or subclavian approaches [1C]
5.4  We recommend that a tunnelled haemodialysis catheter is accessed only by trained dialysis staff (or the patient/carer if supervised or trained) using a strict aseptic approach [1C]
5.5  We recommend an assessment of the exit site and function of tunnelled haemodialysis catheters at each dialysis session [1C]
5.6  We suggest regular dressing changes and routine exit site disinfection, using a solution containing 2% chlorhexidine (or an alternative for those allergic to chlorhexidine) [2C]

## Rationale

### Catheter insertion

Tunnelled haemodialysis catheters are produced by multiple manufacturers and available in a variety of designs. Some are twin catheters, composed of two separate single lumen catheters which are inserted sequentially (eg Tesio-Cath), and others are dual lumen, being a single catheter whose lumen is split into two channels, which separate outside the body into two ports (eg Palindrome, HemoStar, Split-Cath, Permcath). Dual catheters have a number of lumen, tip and side-hole designs, for example curvature or staggered tip openings, which may reduce fibrin sheath formation, catheter thrombosis and recirculation [1].

A small number of randomised trials have compared different catheter designs. In one of the larger studies, 302 patients requiring tunnelled catheter access were randomly assigned to the Palindrome or Hemostar catheter type, with possibly greater 12 month patency seen with the latter (84 v 72%, p = 0.14) [2]. However most studies have shown no difference in infection or patency, and studies have not directly compared dual with twin catheters [3–5]. In the intensive care unit setting, catheter surface coatings (eg, heparin, silver) have demonstrated some short term efficacy in preventing thrombosis or infection, but in haemodialysis settings this approach has not been well studied [6].

The internal jugular vein is most commonly used for catheter insertion, since it has long been known that both insertion complications [7] and central venous stenosis are increased with the infra-clavicular subclavian/ axillary vein) route: for example, in a study of patients with malfunction of established fistulas, prior subclavian vein catheter use was more common in those with subclavian vein stenosis (11/12, 92%) than those with no stenosis (12/35, 34%) [8]. And in a pre-operative venogram study prior to access formation, subclavian vein stenosis was seen in (14/35, 40%) of those with, but none of the 27 without, a current or prior subclavian catheter [9]. A comparative study of 100 patients dialysed either by a subclavian or internal jugular catheter (50 in each group) found stenosis of the subclavian or brachiocephalic vein in 42% of the subclavian catheter group, compared to 10% of the internal jugular group [10]. And a similar study found stenosis in 16/32 (50%) of patients after temporary subclavian catheters versus none of the 20 patients with prior temporary internal jugular catheter [11]. These early studies with both temporary and tunnelled catheters highlight the increased risk of stenosis when using the subclavian vein route, which should therefore be avoided where possible, in particular in children and younger adults, for whom a long term outlook is crucial.

Also, the right sided internal jugular is preferred since the longer and more angulated route from the left internal jugular vein to the superior vena cava, results in a higher risk of catheter malposition, and shorter patency. In a retrospective review of 532 catheters, left-sided catheters were associated with more catheter replacements due to infection or poor flow [12]. However, other factors may reasonably contribute to the choice of catheter location. The longevity of planned or current AV access may be reduced by catheter placement on the same side [13], and it seems logical to avoid the site of pacemakers or other trans-venous devices, though studies have shown that this can be successful in selected cases [14]. It is not clear which of these concerns should take priority, and vessel ultrasound, clinical judgement and patient preference also need consideration.

When conventional locations are unavailable, less common sites, such as the external jugular vein, may also be used successfully [15, 16]. For those with severe central venous stenosis the inferior vena cava may be utilised (discussed further in Chapter 7). To avoid venous stenosis, the femoral (thigh) route is sometimes advocated for tunnelled catheters, though complications such as infection and deep vein thromboses appear more common [17]. Reduced patency is also seen with femoral catheters, perhaps due to repeated bending of the catheter body. In a prospective study of 812 tunnelled catheters, median patency of femoral tunnelled catheters was 116 days, in comparison to right and left internal jugular vein tunnelled catheters, which had respective median patencies of 633 and 430 days [18].

Ultrasound contributes importantly to location selection, since unexpected venous anomalies and thrombosis are common: in a study of 143 patients with a history of prior haemodialysis catheter placement, 26% had jugular vein thrombus, which in 62% of cases was occlusive [19]. Dynamic real-time ultrasound guidance during vein puncture is also preferable, rather than landmark approaches or static ultrasound (used before the procedure but not during). The advantage may seem obvious, and the landmark method is rarely used now in the UK, but a Cochrane systematic review including 7 randomised studies covering 830 haemodialysis catheter insertions compared doppler ultrasound with the landmark method: ultrasound significantly reduced procedure failures, procedure time and complications [20], and these advantages appear to extend to femoral insertions [21]. A subsequent Cochrane systematic review, restricted to internal jugular vein catheter insertions, confirmed these findings and indicated that doppler does not improve on conventional two-dimensional ultrasound [22].

Fluoroscopy is imaging which uses x-rays to obtain real-time dynamic images, allowing direct visualisation of the guidewire, which often must negotiate angulation or stenosis [23], and otherwise may pass aberrantly into the azygous vein. Catheter tip position, which is critical for optimal blood flow, is also visualised: tips should be located within the right atrium (preferably mid-level) since proximal locations encourage fibrin sheath formation and distal locations may lead to arrhythmias, tricuspid regurgitation or inferior vena cava stenosis. In a retrospective study of 532 tunnelled internal jugular haemodialysis catheters, tip position within the right atrium, rather than the superior vena cava, reduced catheter dysfunction, in particular for left-sided catheters [12]. Fluoroscopy seems to reduce misplacement: in a retrospective study of 202 catheter insertions, the addition of fluoroscopy was associated with reduced catheter misplacement (OR 0.13, 95%CI 0.02–0.71) [24], though the advantage may be restricted to left-sided catheters. In another retrospective study of 104 catheters inserted without fluoroscopy, tip malposition (in brachiocephalic or azygous vein) occurred in 6/20 inserted on the left side, but none of the 68 inserted on the right side [25]. Fluoroscopy therefore appears to have obvious advantage at least with left-sided insertions, and has become standard for all catheter insertions in many units.

Catheter conversion (whereby a tunnelled catheter is inserted by wire exchange of a temporary non-tunnelled catheter) has traditionally been avoided by many clinicians, primarily due to infection concerns, though these may be unfounded. In a prospective study of 358 catheter conversions, bacteraemia rates were comparable to de novo insertions (0.8 per 100 days) with similar patency also [26]. Authors note that the location of the temporary catheter, which may be too proximal for optimal tip position and patient comfort, needs to be considered.

Infection is a common catheter-related complication, which is associated with hospital admission and mortality, with risks increased particularly in the early post-procedure period. In most units therefore it has become standard to administer a single dose of prophylactic antibiotic at the time of insertion, either before or after, though high-quality supportive data are hard to find. In one randomised study of 60 haemodialysis catheter insertions, compared to saline placebo, a composite catheter infection endpoint was less frequent in the cefazolin group (1 v 3 events, accurate statistics not reported) [27].

Large studies are only available in non-dialysis settings: in a Cochrane systematic review of 5 trials covering 360 oncology patients having long-term catheters inserted for chemotherapy, prophylactic antibiotics (vancomycin, teicoplanin or ceftazidime compared to no antibiotic) were not clearly associated with protection from Gram positive infection (RR 0.72, 95%CI 0.33–1.58) [28]. These weak/inconclusive studies do not demonstrate a lack of benefit, however, and since adverse effects are rare, and the practice widespread, it seems unlikely that this will be a priority for future research. One may conclude that antibiotic prophylaxis is safe, probably beneficial, and therefore sensible either before or immediately after catheter insertion.

### Catheter care

After insertion, catheter infection remains a constant risk, arising usually from contamination of the external or internal catheter surface, by organisms on the skin of patients or hands of staff. Nursing practices concerning dialysis catheters have evolved to prevent infections, including hand hygiene, aseptic handling technique, exit site dressing changes, and disinfection. Protocols are as much about observation and responsiveness as routine procedure, going hand in hand with prompt detection of exit site abnormalities allowing avoidance or timely treatment of infection. It seems obvious that staff training is key to doing this well, but this is a difficult area for robust studies, since common-sense measures can’t be withheld to prove their worth. This has therefore mostly been studied in the context of quality improvement: ‘before-after’ type studies in which an intervention is studied, often including several individual elements, which aims to further improve existing practice. It is known that staff training can lead to dramatic improvements in compliance with hand hygiene policies [29], but it is more difficult to show effects on clinical endpoints such as infection rates.

For example, one study of a package of nursing interventions in a 70-patient dialysis unit, reported (in conference abstract form) a reduction in catheter-related bacteraemias from 1.1 per 1000 days in the year before the intervention, to 0.1–0.6 per 1000 days in the years after [30]. Some studies have a particular focus on observation or dressing changes: for example, using an observation tool designed to highlight concerning features (redness, oedema, discharge, symptoms - with the mnemonic ‘REDS’) Porazko reported a reduction in exit site infections in a cohort of 40 patients from 0.89 to 0.26 per 1000 days (p < 0.001) [31]. Another study observed introduction of a ‘care bundle’ which included exit site inspection at each dialysis session, with dressings changed if wet, soiled, or not changed in the last 7 days. Catheter-related infections were reduced after introduction of the bundle from 5.7 to 1.1 per 1000 days (RR 0.19, 95%CI 0.06–0.63) [32]. It is not possible to determine which aspect of a multi-component intervention was the most effective, but results consistently highlight the advantage of adequately trained staff, adhering to a defined protocol for catheter care, in achieving low rates of infection.

Beyond staff training, some specific elements of catheter care have been studied separately, including disinfectant types, dressing types, topical antibiotics and catheter locks (agents left in the catheter lumen between dialysis sessions). For exit site disinfection chlorhexidine has largely replaced povidone iodine and sodium hypochlorite solutions. As well as well-established evidence in intensive care settings, studies in dialysis settings are also supportive. One trial compared a protocol involving exit site disinfection using 2% chlorhexidine with a protocol using povidone iodine or sodium hypochlorite, with randomisation at unit level across 422 dialysis units involving around 10 000 patients. Catheter-related infections were reduced by 22% in chlorhexidine units (0.81 v 1.04 per 1000 days, p = 0.02), and benefits appeared to be persistent, though in around 2% of patients local reactions were seen including itching and blistering [33]. To overcome local reactions weaker chlorhexidine solutions have also been assessed in small studies, though 2% is probably superior (RR 0.49; 95%CI 0.18–1.34) [34]. Chlorhexidine may also be superior for catheter hub disinfection [35] though this is less clear, and use of 70% alcohol is also common.

The possibility that occlusive dressings might be improved if impregnated with antiseptic agents seems plausible and has been studied, though largely outside the dialysis setting. A Cochrane systematic review included 22 studies involving 7000 participants with central venous catheters in intensive care units, comparing a number of different dressing designs [36]. Authors found a reduction in catheter-related bacteraemia with chlorhexidine impregnated compared to standard polyurethane dressings (RR 0.51, 95%CI 0.33–0.78), but this outcome in intensive care units, where venous catheter duration is measured in days, may not translate to long-term benefit with dialysis catheters. One before-after study in which dry gauze dressings were replaced with chlorhexidine dressings, introduced in phases across three dialysis units, suggested a modest reduction in infections [37]. But in a crossover trial involving 121 patients, no improvement was seen in the rate of catheter-related bacteraemia, which if anything was increased (RR 1.22, 95%CI 0.75–1.97) [38].

In addition to using antiseptic solutions to clean the exit site when dressings are changed, absorbable antimicrobial ointments may also be applied. These may contain an individual antibiotic, such as mupirocin, or combinations such as Polysporin, which contains polymyxin, bacitracin and gramicidin. Several types have been studied, and the strategy has been studied more generally in a Cochrane systematic review of ten studies [39]. Considering antimicrobial agents collectively (versus no treatment) antimicrobials were effective in reducing exit infection in 4 studies covering 346 patients (RR 0.20, 95%CI 0.09–0.45) and effective in reducing bacteraemia in 5 studies covering 508 patients (RR 0.26, 95%CI 0.15–0.46). Studies included were published between 1991 and 2004 however, and more recent high quality data are lacking. In addition, infection rates in these studies seem high by today’s standards, for example in the HIPPO study Polysporin reduced bacteraemia from 2.48 to 0.63 per 1000 days [40]. A follow-up study reported maintenance of these rates many years later, without evidence of microbial resistance [41], but similarly low infection rates are usually reported with routine care in modern registries.

One further innovation worth discussion is ClearGuard, a novel catheter cap with a chlorhexidine coated tongue which extends around 2 cm into the catheter lumen. In a cluster-randomised study involving 2470 patients across 40 dialysis units, use of this type of cap (discarded and replaced by a new cap each dialysis session) was associated with a lower rate of bacteraemia than standard caps (RR 0.44, 95%CI 0.23–0.83) though the authors acknowledge that not all events were captured, such as bacteraemia occurring outside the dialysis unit in hospital settings [42]. Although promising, these data are highly dependent on clinical setting, and not sufficiently generalisable or compelling therefore for widespread adoption.

At the beginning and end of each dialysis session, the catheter is normally flushed with normal saline to maintain patency, based on the common-sense rationale of preventing fibrin and thrombus build up, rather than evidence. Manufacturers and local non-haemodialysis policies often make recommendations about the size of syringe and use of pulsatile flushing that again are poorly evidenced, but should be adhered to, unless there is an obvious contraindication specific to the haemodialysis setting. At the end of each dialysis session, catheters are usually ‘locked’ with a solution equal to the catheter luminal volume, and intended primarily to prevent thrombosis. The two commonest agents used are heparin, usually at a concentration of 5000 U/ml, and citrate, usually around 5% in studies but higher concentrations (up to 30%) are common in UK practice.

A meta-analysis examined 16 trials comparing citrate with heparin in the prevention of haemodialysis catheter-related complications, between 1998 and 2018 [43]. Comparing citrate with heparin in terms of thrombosis, no difference was found in the requirement for thrombolytic treatment (1.66 v 1.42 per patient year, RR 0.92, 95%CI 0.54–1.57) or catheter removal for poor flow (0.28 v 0.25 per patient year, RR 1.18, 95%CI 0.57–2.44). There was an apparent advantage with citrate in terms of major bleeding complications, though these were not assessed in most studies (4.01 v 7.43 per patient year, RR 0.54, 95%CI 0.33–0.89). There were also apparent advantages with citrate in terms of infection, for example with fewer catheter-related bacteraemias (RR 0.42, 95%CI 0.25–0.69), though the inclusion of studies in which citrate locks were often combined with antimicrobials was probably responsible. In an earlier meta-analysis, when comparing unmodified citrate with heparin, no infection benefit was seen (RR 0.54, 95%CI 0.22–1.30) [44].

The issue of catheter locks incorporating antimicrobial agents was addressed in a Cochrane systematic review of 30 studies involving 3392 patients, with lock solutions containing either an antibiotic (eg gentamicin or minocycline) or a non-antibiotic antimicrobial (eg taurolidine, ethanol or propylparaben) [45]. Analysed as a single group, antimicrobial locks were associated with reduced catheter-related infection (RR 0.38, 95%CI 0.27–0.53), without loss of efficacy against thrombosis (RR 0.79, 95%CI 0.52–1.22). These meta-analyses of catheter locks are somewhat hard to interpret due to the variety of lock types which are pooled as a single group, as well as the differences in concentration.

Systemic treatments which might be effective in preserving catheter function have also been studied, but it is not clear that any is effective. In a randomised trial of 174 haemodialysis patients, low dose warfarin (INR target 1.5–2.0) was not associated with reduced requirement for catheter exchange compared to placebo (HR 0.87, 95%CI 0.42–1.81) [46]. And warfarin was also the subject of a meta-analysis (including this study): in 5 trials covering 479 participants, warfarin was as safe as placebo, but was not clearly associated with reduced catheter dysfunction (RR 0.59, 95%CI 0.28–1.22) [47]. Low dose aspirin (80 mg daily) showed some promise when compared with placebo in an Iranian trial which included 185 patients. Catheter dysfunction requiring exchange occurred significantly later in those taking aspirin (5.3 ± 4.7 v 3.9 ± 2.7 months, p = 0.012) however the short catheter patency in both arms of the study is surprising, and perhaps limits the generalisability to UK practice [48]. No systemic treatment to improve catheter patency can therefore be recommended.

Complications such as infection and dysfunction are more likely when catheters are handled by those unfamiliar with them (which may include non-dialysis clinicians), and air embolism in particular can be the result of incorrect catheter use. Whilst the detail of prevention and management of air embolism is beyond the scope of this guideline, a recent HSIB report on a patient death associated with an air embolus following use of a dialysis catheter by untrained healthcare professionals provides recommendations to mitigate the risk of this complication [49]. Though evidence for the effect of training on complications is limited, the consequences can be serious, so it seems obvious that such an important element of dialysis care should only be carried out by those with adequate training and are deemed competent to do so. Whilst it is common sense to recognise good staff training will prevent these complications, good staff training and procedures will also prevent other rare and unrecognised complications that are also beyond the scope of this guideline to address.

## Guideline 6. Dialysis catheter problems

6.1  We suggest a shared decision in the management of dialysis catheter complications, taking into account clinical severity, treatability, alternative access options and patient priorities [2D]
6.2  We recommend locking each lumen of the catheter with a thrombolytic agent (such as urokinase or alteplase) as the initial treatment for catheter dysfunction [1C]
6.3  We recommend catheter replacement when thrombolytics are ineffective, usually by exchange over a guidewire, in a setting where fibrin sheath disruption is also available [1C]
6.4  We recommend systemic antibiotics without catheter replacement for exit site infections without bacteraemia [1D]
6.5  We suggest systemic antibiotics without catheter replacement as the initial strategy for uncomplicated bacteraemia due to coagulase-negative Staphylococci [2C]
6.6  We suggest routinely favouring catheter replacement, either by exchange over a guidewire or by removal with interval replacement, in the context of bacteraemia which is recurrent, associated with severe clinical features, or due to Staphylococcus aureus [2C]

## Rationale

A proportion of catheters may develop complications over time, of which the most common are dysfunction (poor flow) and infection. As with AV access, much of the literature on catheter complications is limited by small study populations and short term outcomes, leaving many knowledge gaps, so that an evidence-based consensus is not possible for all aspects of management. Since the optimal approach is not always clear, pragmatic shared decisions should be made, taking into account clinical risk, likely treatment outcome and patient preference.

### Catheter dysfunction

Tunnelled dialysis catheter dysfunction is a common problem, usually defined as inability of the catheter to deliver a blood flow of at least 300 ml/min in adult patients. Dysfunction from the time of insertion is generally due to poor positioning or kinking (rare following fluoroscopic guided insertion) and usually identified and corrected soon after insertion, through manipulation or repositioning. A catheter which previously functioned well but then delivers poor flow is considered to have late dysfunction, and this is may be caused by thrombus or fibrin, either within the catheter lumen or around the tip, though the distinction is in most cases not important, and imaging is not necessary. Fibrin forms around the external surface of most catheters, like a sheath, sometimes extending beyond the catheter tip.

If flows become problematic during haemodialysis, then repositioning, saline flushes or reversing the catheter lumens may provide a temporary solution, allowing completion of the session [1]. However, these solutions do not address the cause of poor flow and are rarely durable - further treatment options include thrombolytic agents and mechanical measures (removing fibrin, usually at the same time as replacing the catheter). Thrombolytic therapy is immediately available in the dialysis unit, and is usually attempted initially, as it is often able to restore function quickly allowing dialysis to continue without too much interruption.

Several studies have examined the effectiveness of thrombolytic agents in restoring catheter function, either compared with placebo (one study) or comparing different thrombolytic regimens, summarised in Table 2. These agents are usually administered as a ‘lock’ solution, instilled into each catheter at a volume designed to fill the whole lumen, and remaining there for a period of time (‘dwell’) before being removed (less commonly the thrombolytic is instilled as a ‘push’, in which the dwell volume is supplemented during the dwell by small additional volumes). Though some thrombolytic disperses beyond the catheter, these methods do not deliver much thrombolytic agent systemically, so that adverse effects would not be expected, and indeed no serious adverse events were reported in these studies. In addition, the doses used are small: when used intravenously for pulmonary embolism, for example, up to 100 mg alteplase may be given over 2 hours, and urokinase may be infused at up to 400 000iu/hour over 12 hours.

**Table 2:** Studies of thrombolytic agents for restoring catheter function

Author/country Study design	Inclusion criteria Successful outcome	Treatments	Results
*Studies comparing thrombolysis with placebo*			
Tumlin (2)/USA Randomised N = 151	Flow < 300 ml/min Flow > 300 ml/min	Tenectaplase 2 mg/ml v ‘placebo’ 60 min dwell	22 v 5% success p = 0.004 Favours thrombolysis
*Studies comparing different methods of thrombolysis*			
Pollo (3)/Brazil Randomised N = 106	‘Complete occlusion’ Flow > 250 ml/min	Alteplase 1 mg/ml v Urokinase 5000 iu/ml 40 min dwell	95 v 82% success No clear difference
Donati (4)/Italy Randomised N = 65 (all on warfarin)	‘Thrombotic events’ Flow > 250 ml/min	Urokinase 100 000 iu v 25 000 iu (duration not specified)	100 v 14% success p = 0.01 Favours larger dose
Yaseen (5)/Canada Non-randomised cohorts, N = 237	‘Thrombotic dysfunction’ Catheter durability (time until exchange required)	Alteplase 2 mg v 1 mg (duration not specified)	HR 2.75 p = 0.02 Favours larger dose
McRae (6)/Canada Randomised N = 60	Flow < 250 ml/min (1) Flow > 250 ml/min (2) Flow maintained at 2 weeks	Alteplase 1 mg/ml 1 hour v 48 hour dwell	(1) 77 v 70% success (2) 42 v 53% maintained No clear difference

Only one study (N = 151) compared thrombolytic treatment with placebo [2], demonstrating a clear benefit, with flows restored after a single 60-minute dwell in 22% of patients (v 5% spontaneous improvement with placebo, p = 0.004). Though efficacious, the success rate of a single treatment was low, though generally better in subsequent comparative studies. Another study looked at different thrombolytics, comparing alteplase (1 mg/ml) with urokinase (5000iu/ml), reporting no clear difference, though single-dose success was marginally more frequent with the former (95 v 82%, p = 0.06) [3]. Authors noted also that subsequent doses improved overall success rates in both groups (97 and 88%).

Two studies compared different thrombolytic doses. Donati compared two urokinase doses, in warfarin-treated patients developing catheter dysfunction, favouring the higher dose (100 000iu), though both doses were higher than commonly used, and results in the low dose arm were poor compared to other studies [4]. Different doses of alteplase (2 mg v 1 mg) were compared in a non-randomised cohort study, in which thrombolytics were used as needed over time, with catheter durability (time until replacement) as the main outcome, again favouring the higher dose [5]. Thrombolytic doses in studies are sometimes quoted as concentrations (per ml, so that the per-lumen dose would vary) and sometimes as total dose (per lumen, therefore diluted to reach the correct volume) so they are not easy to compare between studies. However, these results, along with the good safety record for catheter thrombolysis, might reasonably lead clinicians to exceed the lower doses reported in these studies (ie alteplase 1 mg or urokinase 5000iu per lumen).

Dwell time was examined in one study, which compared a 1-hour dwell time with over 48 hours (the whole inter-dialytic interval) of alteplase 1 mg/ml. No clear advantage was seen with the longer dwell, though this is often more convenient for patients than spending an hour in the unit unable to dialyse [6]. Taken together, studies support thrombolytic agents as safe, convenient and usually effective, though repeated treatments may be required. The need for repeated treatment should not be a concern: indeed, routine weekly thrombolytic use (alteplase 1 mg) has been shown to be safe and effective in preventing catheter dysfunction (HR 0.52, 95%CI 0.31–0.88) [7], and whilst this may be insufficient to justify the cost of widespread prophylaxis, it does provide reassurance for using thrombolytic agents liberally in the treatment of catheter dysfunction.

When thrombolytic locks are insufficient, higher dose thrombolytic treatment, delivered over several hours as an infusion, may be successful in restoring catheter function, but this strategy has received only limited study. Gray examined urokinase infusion (250 000iu per lumen over 4 hours) comparing it with fibrin sheath disruption, finding no clear difference in initial success or durability, though both seemed reasonably effective (89 and 97% initial success) [8]. Thrombolytic catheter locks were not used however, so on the question of whether an infusion may succeed where a lock has failed, this study is not informative.

In clinical practice however, most centres take a pragmatic approach based on convenience and safety, using thrombolytic agents initially as a lock, which may be repeated as necessary, escalating to an infusion if this fails. In an observational study of 200 patients with catheter dysfunction in 10 UK dialysis centres, Kumwenda compared urokinase locks (dwell or push) and infusions, given sequentially according to local protocol at various doses, over a 6-month period [9]. Total doses ranged from 12 500iu to 50 000iu for dwell or push locks, and from 100 000iu to 250 000iu for infusions. With a conservative definition (blood flow over 200 ml/min) initial success was around 90%, increasing to 99% with repeated treatments. Infusions were predominantly used after failure of one or more lock attempts, where they were possibly, but not clearly, more efficacious (p = 0.07). Over a 6 month period, 17 patients (9%) had their catheter replaced, in the context of recurrent or persistent dysfunction.

When repeated thrombolysis is unable to restore catheter flow, the catheter is usually replaced, except in situations when suboptimal flow might be acceptable, for example when either prognosis or dialysis requirement is limited. Catheter replacement has the disadvantages of procedural risk and treatment burden, but is usually reliable in restoring flow. Replacement over a guidewire however, which is a common way of simplifying the procedure, sites the new catheter within the same fibrin sheath, if present, so that poor flow may persist after catheter replacement. Disruption of the fibrin sheath under fluoroscopy (usually with a 10 mm angioplasty balloon) eliminates this potential flow problem, and often facilitates catheter replacement also, so this is now reasonably standard when catheters are replaced over a wire. Replacement over a guidewire, when performed for poor flow, should therefore usually be performed in radiology, where any fibrin sheath can be identified and treated. One small study [10] was unable to demonstrate clearly the superiority of this approach (catheter functional for 373 v 98 days, p = 0.22) but since it is safe [11], easy to deliver at the time of catheter replacement, and sometimes necessary anyway, this question is unlikely to attract future research attention. In a small randomised trial, Merport tested the possibility that removing fibrin sheath might be enough to restore flow without changing the catheter [12], but whilst initially successful this method was clearly less durable (25 v 52 days, p < 0.001), and since it is no less invasive, it has largely been abandoned.

### Central vein thrombosis

External catheter-related thrombosis, occluding flow through the central veins, is a less common problem. This may present as face or arm swelling, but is often asymptomatic, found incidentally when imaging is performed for another reason. It can be difficult on imaging to distinguish between thrombus, for which treatment may be considered, and fibrin, which only requires treatment when catheter flow is reduced, but venous dilation by occluding material or the recent onset of occlusive symptoms suggest the former. The main treatment considerations are catheter replacement and anticoagulation. Catheter replacement might improve occlusive symptoms, and allows simultaneous radiological aspiration of thrombus and dilatation of associated stenosis, but it may also precipitate embolisation, so this is usually reserved for catheters which are also dysfunctional. Decisions should consider symptoms, anatomy, comorbidity, and access function, closely liaising with interventional radiology. Evidence is sparse, best summarised in a systematic review of case reports [13], but temporary anticoagulation, for example for 3 months, is usually given to those with symptoms suggestive of an acute event.

### Catheter-related infection

Tunnelled dialysis catheter infections are a significant cause of morbidity and mortality patients undergoing haemodialysis [14]. Three clinical types of catheter infection are recognised: exit infections (defined clinically by the presence of local inflammatory signs or discharge, without systemic illness, and usually confirmed by swab culture); bacteraemia (defined by positive microbiology without another apparent source, though usually suspected clinically in the presence of fever and treated empirically after taking blood cultures, usually from the dialysis circuit) [15]; and tunnel infections (defined clinically by the presence of inflammatory signs overlying the tunnel). Overlapping features may be present, most tunnel infections are accompanied by exit infection or bacteraemia, and blood cultures should therefore be taken before treatment of any catheter infection. The most serious catheter-related infection is bacteraemia, in which in most cases the exit and tunnel are both normal.

Exit site infections without bacteraemia are usually treated systemically for 1–2 weeks, and though recurrence may occur, repeated prolonged treatment is often successful, and catheter replacement rarely needed. Some infections involving the tunnel (but without bacteraemia) may similarly be treated, but clinical judgement is needed, and catheter replacement may often be required for more serious local features such as tunnel abscess or erosion.

Treatment of catheter-related bacteraemia is in some ways consistent between institutions (prompt intravenous broad-spectrum antibiotics, modified by microbiological results, continued for 2–3 weeks minimum) and in some ways variable (catheters may be removed and replaced after an interval, exchanged over a guidewire, or not replaced at all), though catheters are usually replaced in the context of severe sepsis, or when fungi are identified. There are no randomised trials in this area, perhaps in part because of the discontinuity in care between outpatient and inpatient settings, but a number of cohort studies provide some insight.

The most helpful study is a meta-analysis of 28 cohort studies that were published between 1990 and 2013, including 1596 bacteraemia episodes, in which one of three treatment strategies was used: (A) antibiotics alone (N = 697) typically for 3–4 weeks (range 2–6), without catheter replacement; (B) antibiotic lock (N = 546) in which systemic antibiotic treatment is supplemented by antibiotic delivered as a lock between dialysis sessions, throughout the antibiotic period, without catheter replacement; or (C) guidewire exchange (N = 353) in which the catheter is replaced by exchange over a guidewire during the period of antibiotic treatment [16]. Typical antibiotic locks used were vancomycin (2.5 mg/ml), ceftazidime (5 mg/ml) or gentamicin (1 mg/ml), alone or in combination depending on microbiology, added to heparin (5000 u/ml). Treatment strategy selection is not detailed, though it appears to have been largely institutional rather than clinical, and did not appear to depend on the infecting organism, which was distributed roughly evenly between three main groups: Staphylococcus aureus (StA), coagulase-negative Staphylococci (CnS), and Gram-negative bacilli (GnB), with a smaller number of other bacteria or poly-microbial infections.

Cure was defined as clinical resolution without recurrent bacteraemia, over an average observation period of 3 months (range 3 weeks - 6 months), and was achieved in 45%, 57% and 67% of patients in groups A, B and C respectively, with both treatment B (OR 2.08, 95%CI 1.25–3.45) and C (OR 2.88, 95%CI 1.82–4.55) appearing superior to A. This treatment advantage was to a large extent driven by recurrent bacteraemia which was seen in groups A, B and C at 29%, 14% and 7%. Treatment outcomes appeared to interact with organism, with catheter exchange having the strongest advantage in StA infections (OR 4.72, 95%CI 1.79–12.46) which were the hardest to cure, and no clear advantage in CnS infections, which were the easiest. Serious infectious complications, including severe sepsis, metastatic infection and death occurred at similar rates in all treatment groups (9%, 8%, and 8% for pooled rates).

Although the evidence quality is low, one can draw some conclusions from these data to assist in decision making with patients. Bacteraemia, in the absence of severe sepsis (requiring pressors or persisting beyond 48 hours of treatment), may be managed with antibiotics (delivered systemically and via catheter lock) with or without catheter replacement (by either exchange or removal with interval replacement), with replacement being less convenient but more often curative. External catheter appearance, microbiology, and of course patient priorities may contribute to this decision, with replacement usually favoured for StA infections (and by some clinicians for Pseudomonas also). All patients, but particularly those not replacing their catheter, should be aware of the risk of deterioration or recurrence, for which a catheter non-replacement strategy should not be attempted repeatedly. Metastatic infections are not rare complications [17], particularly with StA infections [18], and both treatment duration and monitoring are modified, therefore echocardiography should usually be performed, and other metastatic infections may also be sought depending on clinical suspicion.

## Guideline 7. Central venous stenosis

7.1  We suggest that an awareness of central venous stenosis, including risk factors, clinical consequences and prevention, is important for all clinicians caring for patients with chronic kidney disease [2C]
7.2  We suggest a multi-disciplinary approach to treatment, considering symptoms, access function, patient preference and their kidney replacement therapy journey [2C]
7.3  We suggest that asymptomatic central venous stenosis should managed conservatively [2C]

## Rationale

**Fig. 1:**
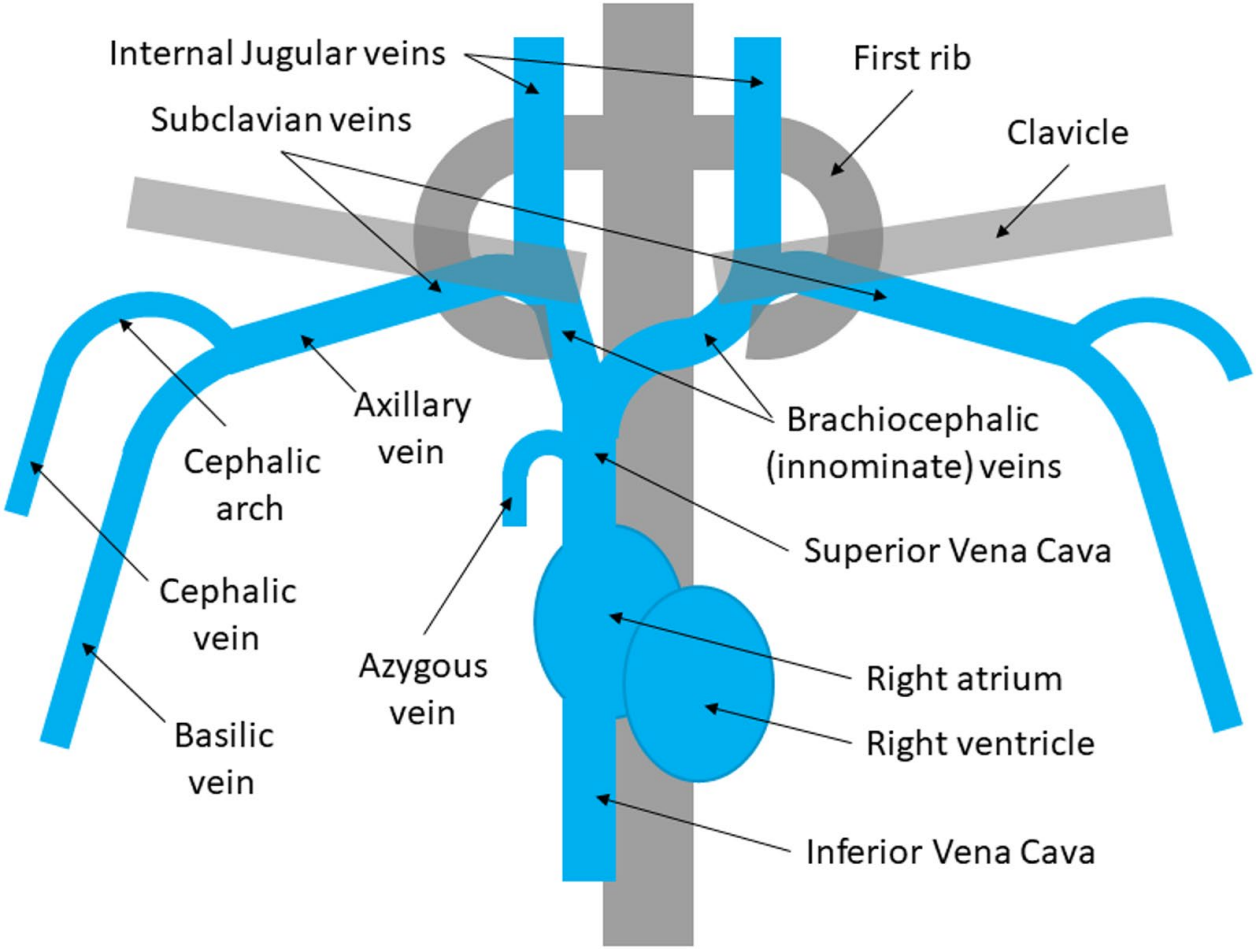
Simplified anatomy of central veins, leading from neck and arms, back to the heart

Central venous stenosis (CVS) is defined as pathological narrowing or occlusion in one or more of the thoracic veins: subclavian, brachiocephalic (innominate) or superior vena cava (SVC), with simplified central venous anatomy illustrated in Fig. 1.

Although it may cause symptoms, such as arm swelling following fistula creation, the clinical importance of CVS is largely due to its effect on access *function*, both success rates and durability. This effect on dialysis access is variable, but severe CVS limits access options, by occluding the necessary outflow for successful AV access formation, and preventing catheter placement: in a minority of cases SVC territory access is no longer possible (see Sect. 4, below). Even when asymptomatic CVS is a hidden cause of access failure, as perhaps best demonstrated in Shingarev’s report of outcomes after fistula formation in 233 patients with a previous dialysis catheter. Comparing patients according to whether the prior catheter was contralateral or ipsilateral to the fistula, although there was no difference in initial fistula success, an ipsilateral prior catheter was clearly associated with shorter secondary patency (HR 2.48, 95%CI 1.33–7.33, p = 0.009) [1].

Diagnosis is usually by contrast venography, though cross-sectional imaging may be helpful. Judged by a venographic gold-standard, CT scanning has been described as specific (97%) but not so sensitive (56%), though sensitivity for symptomatic CVS is likely to be better [2].

Frequency of CVS is dependent on the indication for imaging. It is common in imaging surveys of unselected patients, but most such CVS is clinically insignificant. Studies suggest that clinically apparent CVS affects 5–10% of haemodialysis patients, with a wide range in severity, outlined below. Dialysis catheters, both tunnelled and non-tunnelled, constitute the dominant risk: Adwaney’s study of 500 patients with prior catheter use, described CVS developing in 2% per year, with risk relating to both the number and duration of previous catheters [3]. This, and other studies, also highlight also non-catheter risks including pacemaker wires and external compression. In a large Japanese dialysis program, Kotoda found symptomatic CVS in 26 patients, 19 of whom had never had a dialysis catheter, with 7 cases caused by compression of the left brachiocephalic vein [4]. For unclear reasons, older patients are less prone to developing CVS [3].

Prevention of CVS is one of the key reasons for favouring AV access over catheters, and also provides sound rational for avoiding temporary catheter access, whilst AV access is not mature. The most logical strategy for achieving this is prediction and planning for dialysis initiation or access failure, with prompt referral pathways for assessment and formation. Not all dialysis is predictable however, though even when unplanned, non-catheter options for dialysis are available, with studies reporting the use of early cannulation grafts [5] or femoral (thigh) catheters for emergency access [6], and peritoneal dialysis being an option in some of these settings. For those patients dialysing via catheter access, avoidance of catheter changes, where possible, may limit the development of CVS. Prevention is particularly pertinent in children and young adults, where planning needs to consider a lifetime of kidney replacement therapy. Therefore, for these patients avoiding CVS from dialysis catheters is important (discussed in Chapter 1), with both kidney transplantation and peritoneal dialysis options enabling avoidance of catheters for many children.

Management of CVS depends very much on the clinical setting. Though there is an abundance of literature on the subject, most studies are small case series, subject to selection bias and influenced by local expertise. There is significant heterogeneity in the clinical and anatomical presentations with most studies focussing on one isolated component of treatment, and it can be hard to conceptualise how the multitude of treatment options now available might fit together in clinical practice. As such it is not possible to make clear guideline recommendations regarding most aspects of treatment.

In determining treatment, it is symptoms and access function which matter most, rather than lesion anatomy, though the latter may determine available options. Many other considerations are also relevant, including the expected duration of haemodialysis, feasibility of other modalities and, of course, patient preferences. An approach which considers these factors may be assisted by the concept of a ‘life-plan’ which incorporates ideas of long-term planning, according to patient choices and goals. Promoted in KDOQI guidelines, the life-plan helps teams move away from prescriptive priorities such as ‘fistula first’, towards patient-centred decision making, appropriately recognising vascular access as part of a longer-term kidney replacement therapy journey, which often involves peritoneal dialysis and transplantation. These decisions therefore go beyond access planning, and early multidisciplinary team involvement is key to delivering this well.

In order to summarise the evidence which may guide these decisions, we therefore adopt a clinical classification, which aids the understanding of distinct areas of literature and, for the most part, deals with relevant practical choices. A summary of common treatment approaches and supporting literature according to this classification follows, but it is important to understand that this classification is only loosely related to anatomy, and that categories may not be mutually exclusive.

## Mild CVS: non-symptomatic, with functional access

If CVS is clinically mild (no symptoms, with functional AV access) then no treatment is necessary, and may even be harmful. Most such CVS is not recognised and does not cause any detectable access dysfunction: Shi reported the results of venographic screening in a group of 54 patients with functional AV access, finding CVS in 13 patients (24%), who were no different from others in terms of fistula flow or pressure characteristics [7]. Intervention in such lesions appears to worsen the degree of stenosis [8], and there is therefore no rationale for looking for CVS without clinical indication, or for intervening on an asymptomatic lesion.

It is important to note that the designation ‘mild’ refers to clinical severity, not radiological. Indeed, radiologically occlusive CVS may be clinically mild, due to the development of collateral vessels. Furthermore, even with occlusive CVS, it may be possible to preserve access without symptoms. Jennings described 22 patients with AV access and radiologically occlusive CVS who developed symptoms: they were treated with AV access inflow reduction (without treating the CVS) with full symptom resolution in 20 patients [9].

## Moderate CVS: symptoms or access dysfunction, but easy to open

Moderate CVS refers to cases with clinical features (symptoms or access dysfunction) but without difficulty opening the lesion, though the anatomy and nature of the underlying lesion varies widely between studies. Most studies focus on methods to maintain (rather than achieve) patency and AV access function is usually preserved. The main questions are: (1) how effective is percutaneous transluminal angioplasty (PTA, also called balloon venoplasty), and (2) whether drug coating or stents/stent-grafts add anything to this. The usual study outcome is primary (without re-intervention) patency. Such intervention may treat the CVS lesion itself or another part of the access circuit and, since triggers for intervention may vary between centres, these patency outcomes are only indirectly comparable.

PTA is usually successful in the short-medium term provided there is technical success in overcoming the stenosis, and appears immediately effective in relieving symptoms and improving access function, though re-treatment is often necessary: in 26 patients with AV access (23 left-sided) and CVS, balloon treatment was successfully achieved in 25, resulting in increased access flow (1306 vs 957 ml/min, p = 0.005), with 1-year primary patency 57% [10]. In a larger study of 132 patients with AV access and CVS causing symptoms or access dysfunction, 1-year primary patency with balloon treatment was 74% [11].

The effect may be more durable if venoplasty balloons are coated with an anti-proliferative drug, such as paclitaxel. Kitrou randomised 40 patients with AV access and symptomatic CVS to venoplasty with paclitaxel-coated or standard balloon, demonstrating longer median primary patency in the paclitaxel group (6 vs 4 months, p = 0.03) [12]. Given the small sample, modest effect size and relatively rapid recurrence of the target lesion in both study groups, this is promising rather than conclusive, with both treatment time and cost increased.

Stents are regularly employed to combat the rapid elastic recoil that often follows balloon venoplasty, and they probably also improve the durability of CVS treatment. Stents are sometimes bare metal, though increasingly covered stents (also called ‘stent grafts’) are used, and whilst most studies separate types, a few treat both types of stent as a single group: for example, Shi demonstrated superior 1-year patency of ipsilateral AV access in patients with symptomatic CVS, treated with either stent type versus balloon-only, though the difference may have been due to chance (49% vs 77%, N = 24, p = 0.20) [13]. But in a meta-analysis of 8 studies including 281 balloon-only treatments and 192 stents of either type, no clear difference was found [14].

Covered stents have clearer support in the literature: a number of cohort studies have described their use in the context of CVS, achieving 1-year patency ranging from 40% (N = 52) [15] to 88% (N = 60) [16]. In addition, central veins formed the largest location subset (35%) in a study of AV access stenosis within previously placed bare metal stents at any site (N = 275, 54% fistulas, 46% grafts), randomly allocated to treatment with covered stent or balloon-only treatment [17]. In the whole study, target lesion primary patency at 6 months was better with covered stents, with this benefit also clearly seen in the CVS subset (12-month CVS primary patency 30 v 4%, p < 0.001).

Bare metal stents have been studied less frequently, though they also appear favourable compared to balloon-only treatment: for example Gur observed 150 patients undergoing treatment for symptomatic CVS with ipsilateral AV access, achieving technical success with 141 (32 stents and 109 balloon-only). Improved primary patency at 1 year (59 v 42%) and at 5 years (28 v 20%) was seen with bare metal stents compared to balloon-only treatment (p = 0.036) [18]. Where both stent types have been compared however, covered stents appear more favourable: for example Quaretti observed 70 patients undergoing treatment for symptomatic CVS, split evenly between those with AV access or a catheter. Technical success was achieved in all, but primary patency at 12 months was 100, 80 and 58% after covered stent (N = 20), bare metal stent (N = 28), and balloon-only (N = 22) treatment respectively (p = 0.020 for covered stents v others) [19].

It should be noted that in uncontrolled studies, stents are primarily used when there is rapid recoil or early recurrence: this introduces a distinct indication bias favouring balloon-only, so it is likely that stents are offering benefit in these cases, and there is sound rationale for their use in recurrent or resistant disease. Pragmatically therefore, in the treatment of central venous stenosis which can be successfully crossed with a guidewire, balloon angioplasty is the modality favoured by most clinicians, with stents reserved for cases of recoil stenosis or early recurrence. Bare metal stents appear to provide no advantage in terms of patency compared to balloon angioplasty alone, but covered stents seem more promising with data from many retrospective studies suggesting they provide a more durable solution, though further studies are awaited to clarify this benefit.

## Severe CVS: difficult to open, access usually dysfunctional

In severe CVS symptoms are very common in the presence of ipsilateral AV access, but variable with catheter access. These lesions are usually hard to open and most studies focus on the method used to cross the lesion with a wire, with fewer focussing on subsequent access.

### Opening the lesion

In most studies of this type of CVS, the focus is on successfully opening the lesion by first crossing it with a wire (so that the lesion can be dilated), often termed recanalisation, though some reserve this term for lesions which initially appear occlusive. The distinction is perhaps unimportant since ‘occlusive’ is only really determined after failure to (or a decision not to attempt to) open the lesion, but the level of radiological difficulty varies, and studies are therefore not really comparable. For non-practitioners these procedures may be hard to understand, and a detailed review is outside the scope of this guideline, but we outline a few studies covering briefly the main ‘non-standard’ techniques. The focus is largely on achieving access in the short term, and subsequent access durability is usually not assessed.

Co-axial catheter systems are used in coronary intervention, and may be useful in opening CVS lesions. For example, Wan reported their use in 45 patients with ‘occlusive CVS’, achieving success in 43 (96%) [20].

Bi-directional approaches (sometimes termed ‘through and through’ or ‘flossing’) in which neither wire can be advanced but one can be snared from the other side and pulled through, are often successful when neither uni-directional approach has been. As examples, Huang reported 25 of 30 successful [21], and Yang reported 14 of 16 successful, though with two minor cases of haemopericardium, and one fatal arrhythmia [22].

‘Sharp recanalisation’ is performed using a needle (for example trans-septal needles, which are used to cross from right to left atrium during arrhythmia ablation procedures). This has been reported as successful in 13 of 16 patients [23], and 12 of 16 patients, though in the latter study the remaining 4 were all achieved at a second attempt [24].

‘Inside-out’ recanalisation is a novel method in which some right sided-central venous lesions can be opened from the femoral route using the Surfacer device (developed by Bluegrass). Access is obtained via the right femoral vein and under radiological guidance a stiff but blunt sheath is passed via the inferior vena cava (IVC), right heart and superior vena cava (SVC) which may be partly occluded. A needle wire is then advanced to exit the skin via the occluded right internal jugular vein, facilitating antegrade access to the central vessels. Several recent studies have demonstrated good success rates: for example Reindl-Schwaighofer reported a multi-centre study of 39 procedures (36 for lesions without SVC involvement) of which 38 were successful, with no early complications [25]. One study (of 10 patients) reported one early post-operative death [26], but similar success (27 out of 30), again without complications, was found in a prospective study [27].

### Subsequent access

A common access after treatment of severe CVS is a catheter through the lesion. Subsequent symptoms are uncommon and the catheter facilitates opening the lesion in the event of a requirement for retreatment. Patency of catheters in this setting is sometimes reported to be similar to other catheters, for example the 77% 1-year patency reported by Huang in 30 patients after bi-directional lesion treatment [21]. Other studies report slightly shorter patency than catheters without CVS: in a single centre study Adwaney observed 176 catheters placed through a stenosis after balloon dilatation, finding a median patency of 20 months [3].

De-novo AV access is not usually attempted in the presence of severe CVS since complications and early failure are common. In a report by Jennings, 19 patients had upper limb fistula formation with known occlusive CVS but extensive collaterals. Symptoms of CVS were seen in 8 patients, with 6 requiring intervention [28]. However, one innovation worth mentioning is the HeRO graft, which uses a catheter to maintain CVS patency at the same time as preserving AV access.

The HeRO (Hemodialysis Reliable Outflow) graft is a conceptually novel form of AV access in which an AV graft is connected at the venous outflow to a silicone-based catheter extension which passes through the stenotic central veins into the right atrium. The device is dependent on the ability to open or bypass a severe CVS lesion, and may be used to provide de novo access or salvage a failing fistula. In the first clinical study Katzman reported HeRO graft placement in 38 patients, of which 36 were successful [29]. Over a mean observation of 9 months, secondary patency (in successful grafts) was 72% with a re-intervention rate of 2.5 per year, but adverse outcomes were frequent including bacteraemia (17), arrhythmia (3), heart failure (3), and death (13). Complications were independently reviewed, with only 7 bacteraemias, and none of the deaths adjudicated as ‘probably or definitely device-related’. Device-related pulmonary embolism occurred in one patient, and pulmonary emboli have also been reported as complications of HeRO grafts in several other series [30, 31].

Subsequent studies have reported 1-year secondary patency ranging from 30 to 91%, with complications which are less frequent but still noticeable. For example, in a multi-centre UK study which included 52 patients, Hunter reported 1-year secondary patency 77% (95%CI 65–91%) with a re-intervention rate of 2.3 per year, and complications including infection (4) and steal syndrome (2) [32]. In a meta-analysis of 8 studies, Al Shakarchi summarised access outcomes after 409 HeRO graft insertions, finding 1-year secondary patency 59% (95%CI 39–78%) with a re-intervention rate of 1.5–3.0 per year [33]. Other than bacteraemias (rate 0.1–0.7 per 1000 days) complications were not assessed in this study.

## Occlusive CVS: non-SVC access required

This section deals with thoracic CVS which is bilaterally occlusive, so that non-thoracic access is therefore required. Occlusive is a variably used term, since clearly some CVS is described as occlusive but then is still opened, and this possibility may be dependent on local expertise and patient preference. We use the term when SVC territory access has been abandoned (which may be the case even with non-occlusive CVS), with studies in this section focussing on non-SVC access.

Symptoms are variable, with the dominant clinical challenge of being able to achieve durable dialysis access: catheters in the inferior vena cava (IVC) territory, thigh grafts and thigh fistulas are the most studied access types.

Catheters in the IVC territory include those inserted into femoral (thigh) veins, hepatic (liver) veins, or directly into the IVC (lumbar catheters), usually under CT guidance. Several studies describe their outcomes, which range from slightly inferior to equivalent to catheters in the SVC territory: for example, Power reported 1-year secondary patency 73% (median patency 18.5 months) following 39 procedures, with a bacteraemia rate of 0.8 per 1000 days [34]. Jonszta also reported outcome in 39 IVC catheters, describing secondary patency 89% at 1 year, no different from patency with 196 catheters in the internal jugular vein [35]. Femoral catheters are generally less durable, but similar outcomes are achieved with the hepatic route. Centre expertise and preference seem to be the main determinants of practice [36].

AV access with IVC territory outflow includes thigh grafts and thigh fistulas, with the former most traditionally performed. Outcomes were historically disappointing, but more recent studies suggest this option deserves reappraisal. Han described thigh graft (common femoral artery to femoral vein) outcomes in 67 patients, reporting 79% secondary patency at 1 year, with 1.8 re-interventions per patient over a median of 50 months [37].

Lower limb fistulas can also be fashioned from either long saphenous or deep femoral veins. Bourquelot reported 72 thigh fistula formations (by femoral vein transposition) which achieved 84% secondary patency at 1 year [38], though it should be noted that 13 patients experienced severe complications necessitating fistula ligation, including one below-knee amputation. Others have reported similarly favourable 1-year secondary patency outcomes from transposed femoral vein fistulas: 95% in 21 patients [39] and 93% in 18 patients [40], with a re-intervention rate of 0.4 per patient-year.

In one of the few comparative studies, Aitken described 127 vascular access formations in 62 haemodialysis patients with occlusive CVS [5]. In terms of both 1-year secondary patency and bacteraemias per 1000 days, the most favourable access type was the saphenous vein fistula (78%, none) though it was not always achievable and the number was small (N = 15). Thereafter the most favourable access type was IVC catheter (1-year secondary patency 50%, bacteraemias per 1000 days 0.6, N = 25), followed by thigh graft (42%, 1.6, N = 25) and femoral catheter (28%, 1.8, N = 62).

The same authors also noted that, where it could be achieved in the patient group, peritoneal dialysis (N = 8) and priority transplantation (N = 11) both gave favourable outcomes. Alternative kidney replacement modalities should be considered alongside vascular access planning for patients with CVS. Transplantation in this setting, either through wait-list priority or live donation, reduces both the number and duration of hospital admissions [41].

### Unconventional and experimental options

A number of less common approaches have also been described with reasonable outcome, though one should remember that small studies of uncommon treatments are particularly prone to publication bias. These techniques may quite reasonably be offered in selected cases, but more conventional methods should generally be preferred, and institutions should be encouraged to prospectively audit access outcome with these less common methods, including failed attempts.

1. Catheters have been surgically inserted directly into the right atrium (not via SVC or IVC). However, complications are frequent, and studies, many of which are case reports, report patient survival rather than access survival: in a systematic review of 51 cases, median patient survival was 25 months [42].
2. Non-thigh grafts have been described for occlusive CVS, with a range of outcomes. For example, Jakimowicz reported a series of unconventional graft placements, including 30 with SVC territory outflow for unilateral venous occlusions, but also 19 axillo-iliac grafts for occlusive CVS, achieving 96% secondary patency at 1 year [43].
3. Arterio-arterial (AA) access has been described, in which initial outflow is non-venous, and thus less impacted by CVS. For example, Khafagy described prosthetic brachial AA loop formation in 35 patients, achieving 91% secondary patency at 1 year [44].

## Data Availability

No datasets were generated or analysed during the current study.

## Appendix A: Glossary of medical terms used in the guideline

**Access**. The device used to connect a patient to the dialysis machine (fistula, graft or catheter).

**Air embolism.** A sudden life-threatening condition due to air in the circulation, which may be introduced by faulty or misused dialysis access or tubing.

**Anaesthesia.** Putting a person, or part of the body, to ‘sleep’ to allow an operation. There are three main types:

- **Local anaesthesia (LA).** An injection is used to numb the area of the operation. Only small areas can be treated this way.
- **Regional anaesthesia (RA).** An injection is used to block the nerves supplying part of the body (e.g. the arm).
- **General anaesthesia (GA).** The patient is unconscious during the operation.

**Anastomosis**. A surgically made join between two things, which in this guideline is used to describe the join between artery and vein, in a fistula.

**Aneurysm**. An unusually wide part of an artery or fistula.

**Angioplasty**. A treatment delivered to a blood vessel on the end of a wire (e.g. stretching open a tight bit of the vessel with a balloon).

**Area Puncture.** This is a needling technique, where the needles are inserted into a similar place each time. Needle sites cover a small area and are unplanned.

**Arteriovenous (AV)**. Describing something which connects or involves an artery and vein

**AV access.** A fistula or graft

**Buttonhole.** A needling technique that involves inserting the needles in exactly same place, in exactly the same way each time. It involves removing the scab from the previous cannulation before inserting the needle into the same hole. Gradually a track of scar tissue is developed leading to the vein and once this happens, blunt or dull needles can be used. Buttonhole normally involves having 2–4 different needling sites.

**Cannulation**. The insertion of needles (e.g. into a fistula or graft), which is also called ‘needling’.

**Catheter**. A tube placed into a large vein which is used to connect a patient to the dialysis machine. Short term catheters enter the skin and vein in the same place. Long term catheters are ‘tunnelled’ entering the skin and vein in different places, (more secure and less prone to infection).

- **Tip**. The internal end, deep inside a large vein, close to the heart.
- **Hub.** The outside end, connected to the dialysis tubes, or covered with a casp when not in use.
- **Exit site**. The hole in the skin where the catheter enters the body.
- **Tunnel**. The part of the catheter which is under the skin but not in the vein. You can usually feel this part as a ridge under the skin, going from the exit site up to the collar bone.

**Central vein**. Veins in the chest, leading back to the heart

**Central venous stenosis (CVS)**. A narrowing of a central vein

**Coagulase-negative Staphylococci (CnS).** A kind of bacteria.

**Comorbidity**. Long term illnesses other than kidney failure which a patient may have permanently, like diabetes.

**Computed Tomography (CT).** A type of scan which produces cross-sectional pictures (like slices through the body).

**Failure.** Permanent loss of function of an access. This may described as:

- **Primary failure.** Loss of function without ever using the access successfully for dialysis.
- **Secondary failure.** Loss of function of an access which was previously functioning normally.

**Fistula**. A connection between and artery and a vein, which makes the vein get bigger, with a faster blood flow. Two needles are inserted into the vein to connect a patient to the dialysis machine, and are removed at the end of the dialysis session. Different parts of a fistula may be described in different ways:

- **Proximal**. Nearer to the head.
- **Distal**. Further away from the head.
- **Upstream**. Nearer to the origin of flow.
- **Downstream**. Further along the direction of flow.

**Fistuloplasty**. A treatment delivered to the fistula on the end of a wire, inserted through a needle. Usually stretching open a tight bit of the fistula with a balloon.

**Fluoroscopy.** A dynamic kind of x-ray which allows multiple pictures over time. When contrast is injected this is used to see blood vessels.

**Glomerular filtration rate (GFR).** A commonly used measure of kidney function, similar to a percentage, calculated from creatinine, a blood test.

**Graft.** Like a fistula but made with a ‘plastic’ tube used to mimic a vein. This is often made of PTFE, a kind of non-stick polythene which blood doesn’t usually clot against.

**Gram-negative bacilli (GnB).** A kind of bacteria.

**Haematoma**. A large pool of blood in the wrong place, resulting from an internal bleed, often leading to a bruised appearance on the skin, though the skin may appear normal if the haematoma is deep.

**Haemorrhage**. Bleeding, usually the term is used for bleeding outside the body.

**Heart failure (cardiac failure)**. A weakness of the heart, so that blood is pumped less strongly. This commonly leads to fluid retention and breathlessness, though kidney failure may also cause these same symptoms.

**Inferior vena cava (IVC).** The large vein just below the heart.

**Intercurrent illness**. Short term illnesses (other than kidney failure) which a kidney patient may develop and get better from, like a chest infection.

**Kidney Disease Outcome Quality Initiative (KDOQI).** A program of education and guideline production run by the National Kidney Foundation (a non-profit American health organisation).

**Mortality**. Statistical term for the occurrence or timing of death in a population.

**Morbidity**. Statistical term for symptoms and illness but not death.

**Neuropathy**. Nerve damage. Occasionally this arises after fistula formation, due to the disruption in blood flow rather than directly due to the operation. This may cause weakness or loss of sensation, and may get better slowly or be permanent.

**Patency**. The length of time for which the access is patent (working rather than blocked).

- **Primary**. The time until the first procedure needed to keep the access working.
- **Secondary**. The time until the access stops working permanently and is abandoned.

**Percutaneous transluminal angioplasty (PTA).** A method of dilating a blood vessel using a balloon on the end of a wire, inserted through a needle, using x-ray to check the position.

**Pseudoaneurysm**. A leak in the fistula, going outside the fistula into the tissues. Sometimes hard to distinguish from an actual aneurysm, in which there is no leakage, but the vessel is bigger in one area.

**Rope Ladder**. This is a needling technique where the needles are inserted 5–10mm above the previous needle sites. Needle sites progressively move up the vein and once the tope is reached, needling starts at the bottom again.

**Peripheral vein**. Veins in the arm or leg.

**Saphenous vein.** A large vein in the leg, which can be surgically removed and then used elsewhere.

**Staphylococcus aureus (StA).** A kind of bacteria.

**Steal**. A reduction in blood flow to the hand, caused by the diversion of blood into the fistula (as if blood is being ‘stolen’ from the hand by the fistula), making the hand cold or painful.

**Stenosis.** A narrow or tight bit of a fistula or vein. This often causes low flow and poor dialysis, and may lead to thrombosis.

**Stent**. A small metal mesh placed permanently inside a vessel to hold it open after stretching it, so it doesn’t get tight again.

**Superior vena cava (SVC).** The large vein just above the heart.

**Thoracic**. In the thorax (chest).

**Thrombosis**. A blood clot which may block a fistula or graft, stopping it from working. An operation may be able to remove the clot and get the access working again.

## Appendix B: Understanding study descriptions and statistical terms used in the guideline

### Terms describing types of study

**Quantitative.** A type of research study that assesses outcomes of a treatment or disease, that uses numbers to provide results. Cohort studies and trials are types of quantitative research.

**Qualitative.** A type of research study where the words of participants are combined to learn about their opinions or lived experience of something. This type of research is often used to uncover patients’ experiences of a treatment.

**Mixed Methods.** A type of research study that combines both quantitative and qualitative designs. It’s often used to assess more than one perspective of the intervention that is being studied. Surveys are often, though not always, mixed methods studies.

**Case report or case series**. A description of a single patient or small group of patients, usually to illustrate a rare condition or novel treatment approach.

**Survey (cross-sectional study)**. A study involving patients at a single time-point only.

**Cohort study**. A study which follows a group over time, for example looking at survival.

**Trial**. A study which recruits patients having a treatment, to study the effect of the treatment.

**Controlled trial**. A trial which compares two groups, either treatment A v treatment B, or treatment v no treatment.

**Placebo**. A tablet with no active ingredient, or more generally the name of the ‘no treatment’ group of a study.

**Randomised controlled trial**. A controlled trial in which allocation of participants to groups is random (in theory balancing out characteristics, even unknown ones, so that the treatment is the only difference between groups). Results are only valid for outcomes which have been pre-specified. Results which were not specified beforehand but which authors still wish to report are known as ‘post-hoc’ outcomes: these are less secure and need cautious interpretation.

**Systematic review.** A type of research study that examines the results of all studies on one intervention or phenomenon. The results of studies are combined to provide an overall result, which provides more confidence in the study results.

**Meta-analysis**. A statistical technique that combines the results of different studies, to provide a pooled estimate of the effect of a treatment, from different studies examining the same treatment. This often gives a more confident estimate than one study on its own. Meta-analysis is usually combined with a systematic review.

### Terms which quantify the effects of disease and treatments

**Significance**. How sure we can be that this effect is ‘real’ and not just a chance observation. This depends mostly on the size (number of participants) of the study. For example, if you toss a coin 5 times, and get 4 heads (80% heads), that doesn’t mean its a weighted coin (with a fair coin the chance of getting at least 4 heads from 5 tosses is 9%, so no big deal). But if you toss it 50 times, and get 40 heads (still 80% heads), it almost certainly is (with a fair coin the chance of getting at least 40 heads from 50 tosses is about 1 in 100 000). A ‘significant’ result is one which is unlikely to be due to chance.

**P value**. The probability (likelihood) of getting the result we observed purely by chance. In other words: if the treatment doesn’t actually make any difference, how likely is it we could see this result? This helps determine significance. A p value equal to 0.02 means that if the effect isn’t real (e.g. if treatment A isn’t actually any better than B), there would be only a 2% chance of getting a result as extreme as we observed. A p value less than 0.05 is often used as the cut-off below which one can describe the result as ‘significant’.

**Risk factor**. Any characteristic which might alter the risk of an event, like smoking, having diabetes, or having a particular treatment.

**Relative risk (RR)**. The ratio of two probabilities, ie. the chance of an event (e.g. getting cured) with treatment A, divided by the chance of the same event with treatment B. If RR = 2, then treatment A makes the event twice as likely.

**Odds ratio (OR)**. The ratio of two odds. Odds and probability are similar but not quite the same - the probability of getting heads on a coin toss is 1/2 (= 0.5 or 50%) often therefore called ‘50:50’, whereas the odds of getting heads is 1/1 (=1) often called ‘even odds’. Probability is more intuitive for most people, but odds can multiplied and behave ‘nicely’ in mathematical terms so they are often preferred in statistical analysis. But you don’t normally need to worry about the difference when interpreting study outcomes.

**Hazard ratio (HR)**. Similar to odds ratio except that the interest is *when* the event occurs rather than *if*, like death for example. Usually reported alongside percentages of participants reaching the event by a particular time (e.g. a year), or median (average) time before the event occurs.

**95%CI (95% confidence interval).** An error-range within which we are 95% sure that the ‘true’ effect size lies. More useful than p values, and often provided instead, confidence intervals which don’t contain 1 are ‘significant’ (ie. the p value is less than 0.05). The confidence interval gives a sensible range for an effect which has been estimated, but is not accurately known.

### Other statistical concepts

**Bias**. Any way in which a study is not ‘fair’. This is not the same as ‘noise’ (unknowns which make the true effect hard to see) because bias refers to things which tend to push the effect artificially in one direction.

**Confounding**. Associations which lead to bias. These may be known, like age or comorbidity, in which case you can attempt to correct the bias by ‘adjusting’ the analysis (which works but not perfectly). But they may also be hidden, in which case you can only guess what they are and how biased the result is. One of the reasons to randomise treatments in a study, is that even hidden factors should then be balanced. Important types of confounding are:

- **Ascertainment**. Study participants who receive the treatment, may be observed more closely than those in the placebo group, so perhaps headache might be more often recorded, just because those patients are seen more often. It would appear as though the treatment causes headache.
- **Selection.** If treatments are compared by observation (just looking at those who did versus didn’t receive a treatment, for whatever reason) rather than in a trial, then the reason for selecting treatment is an important confounder. Sicker patients often opt for simple treatment or no treatment at all, so those who weren’t treated often have poorer outcome anyway, which has nothing to do with the treatment itself.
- **Intention to treat.** When comparing treatments by observation, it is often only *successful* treatments which count as the treated group, those with unsuccessful treatment end up counted in the ‘no treatment’ group (‘as treated’ analysis). Then the factors which made treatment unsuccessful (like being older) get interpreted by mistake as the effect of not having treatment, and the treatment looks better because failed treatments aren’t counted.

**Noise.** The ‘random’ or meaningless variation in data, which can be hard to distinguish from the ‘signal’ (the effect we are interested in), and reduces the accuracy of an estimate. Noise may arise from imperfect measurement tools, human error, and things which can’t be predicted. Noise is different from bias in that it doesn’t push the result in a particular direction, and can usually be reduced (relative to the signal) by doing a large enough study.
